# Emerging 3D bioprinting applications in plastic surgery

**DOI:** 10.1186/s40824-022-00338-7

**Published:** 2023-01-03

**Authors:** Pu Yang, Yikun Ju, Yue Hu, Xiaoyan Xie, Bairong Fang, Lanjie Lei

**Affiliations:** 1 Department of Plastic and Aesthetic (Burn) Surgery, The Second Xiangya Hospital, Central South University, Changsha, 410011 People’s Republic of China; 2 School of Clinical Medicine, North Sichuan Medical College, Nanchong, 637000 People’s Republic of China; 3 Department of Stomatology, The Second Xiangya Hospital, Central South University, Changsha, 410011 People’s Republic of China; 4 School of Biological Science and Medical Engineering, Southeast University, Nanjing, 210096 People’s Republic of China

**Keywords:** Plastic surgery, Biomaterials, 3D bioprinting, Tissue engineering, Tissue regeneration

## Abstract

Plastic surgery is a discipline that uses surgical methods or tissue transplantation to repair, reconstruct and beautify the defects and deformities of human tissues and organs. Three-dimensional (3D) bioprinting has gained widespread attention because it enables fine customization of the implants in the patient's surgical area preoperatively while avoiding some of the adverse reactions and complications of traditional surgical approaches. In this paper, we review the recent research advances in the application of 3D bioprinting in plastic surgery. We first introduce the printing process and basic principles of 3D bioprinting technology, revealing the advantages and disadvantages of different bioprinting technologies. Then, we describe the currently available bioprinting materials, and dissect the rationale for special dynamic 3D bioprinting (4D bioprinting) that is achieved by varying the combination strategy of bioprinting materials. Later, we focus on the viable clinical applications and effects of 3D bioprinting in plastic surgery. Finally, we summarize and discuss the challenges and prospects for the application of 3D bioprinting in plastic surgery. We believe that this review can contribute to further development of 3D bioprinting in plastic surgery and provide lessons for related research.

**Graphical Abstract:**

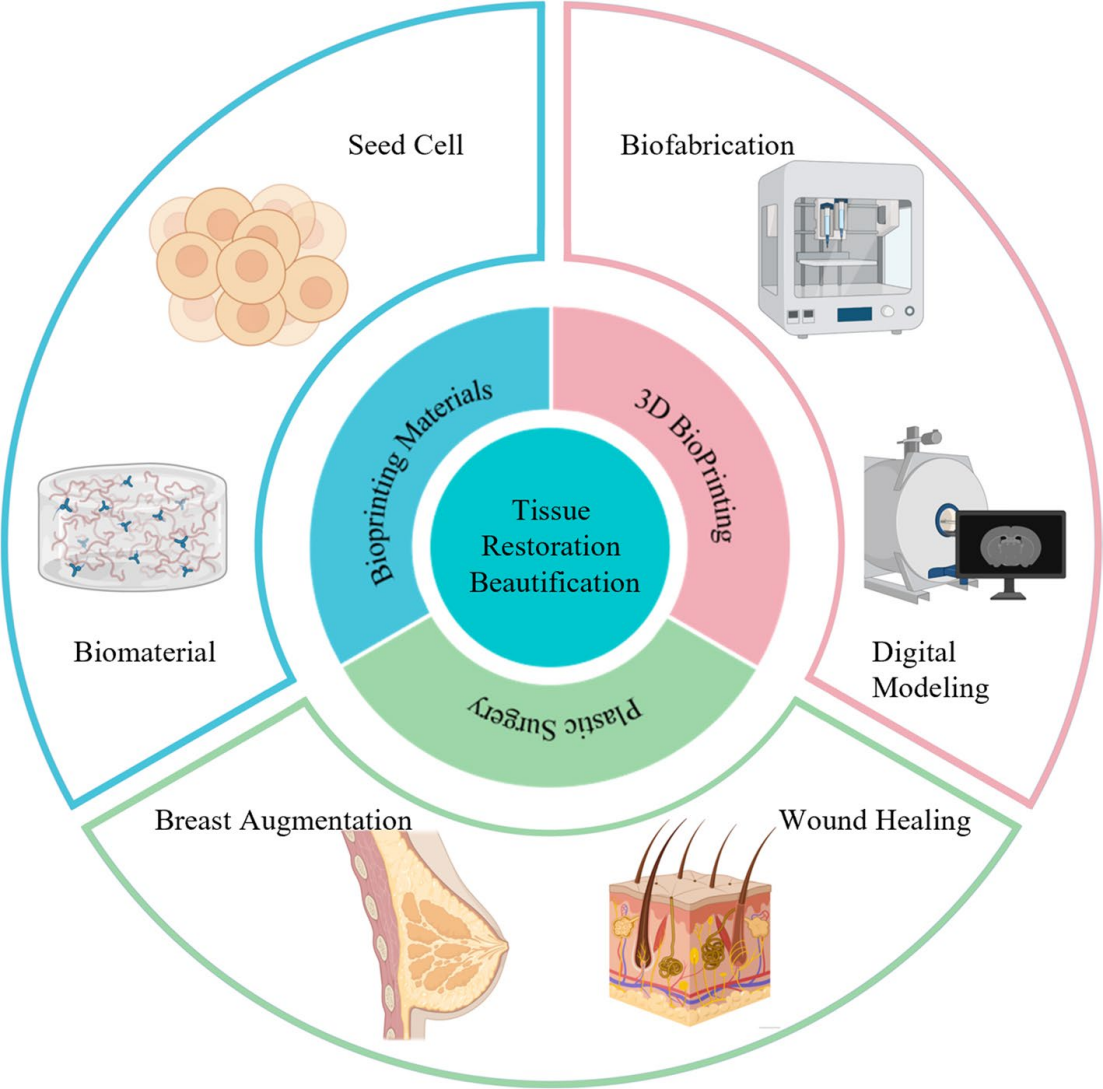

## Background

Tissue and organ damage or deformity due to disease, trauma, tumors, congenital malformations, and other factors place a huge physical and psychological burden on patients. The restoration and beautification of patients' tissues and organs has long been a problem that plastic surgeons have struggled with, but traditional surgical treatments often have limited capabilities. In clinical surgical treatment, it is often necessary to design implants with specific shapes for different patients in order to achieve the aesthetic requirements of the patient. Traditional biomaterials (e.g., expansion, silicone, etc.) often fail to meet the individual requirements for high precision, and may also result in postoperative complications, such as encapsulation, infection, and bleeding. However, autologous tissue transplantation introduces the problems of donor site damage and increased surgical difficulty [1–5].

3D bioprinting technology allows the design of individualized grafts for each patient's needs, resulting in a higher degree of precision and fit. Thus, it provides significant reduction in the difficulty, risk, and duration of plastic surgery. With the development of Three-dimensional(3D) bioprinting technology, various new types of bioinks and printing strategies have emerged, making it possible to customize personalized grafts. Implants prepared using biomaterials and printed based on the patient's own seed cells have superior biocompatibility and lower immunogenicity than conventional biomaterials. Moreover, surgical complications such as pain and inflammation caused by surgical manipulation of the donor area during autologous transplantation are avoided [6–12]. In addition, novel printing technologies such as intraoperative bioprinting have provided new ideas for the clinical practice of plastic surgery [13].

In this review, we focus on the use of 3D bioprinting in plastic surgery. First, we briefly introduce the basic principles, process, advantages, and disadvantages of each type of 3D bioprinting technology. We also describe in detail the currently available bioprinting materials for different types of tissue repair. Moreover, we present 4D bioprinting technology achieved by changing the combination strategy of bioprinting materials, most of the current bioprinting products differ in functionality by changing the structure of the printed product and the composition of the bioink/biomaterial ink. Next, we highlight the specific applications of 3D bioprinting in plastic surgery. And finally, we briefly discuss the current challenges and future prospects of 3D bioprinting in plastic surgery research and application.

## Main text

### 3D bioprinting manufacturing

#### Definition of 3D bioprinting

3D bioprinting based on 3D printing technology that prints cells or other biomaterials on a substrate through a printing system according to the requirements of bionic morphology, organism function, and cellular microenvironment [14, 15]. This delicate process ensures that individual cells or multiple cell types are held together when formulated into biocompatible materials, forming biologically functional 3D constructs. Moreover, bioinks based on cellular components are prepared into shapes according to printing needs, so that the final printed product has a complex geometry, thereby creating various types of 3D biomimetic constructs. Thus, this technology makes it possible to print functional cell-based tissues or organs [16–19].

Specifically, 3D bioprinting work is usually divided into three main steps: pre-processing, processing, and post-processing. 1) Pre-processing: digital imaging and communications in medicine (DICOM) images obtained by segmenting the tissues and organs at the target regionlayer by layer using computed tomography (CT), magnetic resonance imaging (MRI), and other imaging techniques. The obtained images are reconstructed to obtain the 3D model. Then, the 3D model is converted to standard tessellation language (STL) digital language form. 2) Processing: The staff extracts seed cells from the patients, then cultures and proliferates them in vitro*,* then mixes the seed cells with bioink having similar biological properties to the target tissue and configuring the mixture into the printer cartridge. Then, the obtained STL data is used to print out the tissue or organ. 3) Post-processing: Before transplanting the printed product into the tissue of the patient or test model, it should be placed in a bioreactor to maintain its mechanical properties and biological functionality [20, 21].

Since bioprinting was proposed in the early 2000s [22], this technology has developed rapidly and has received widespread attention from research scholars. During the decades of rapid technology development, three main types of mainstream 3D bioprinting technologies have emerged: extrusion-based, droplet-based and laser-based bioprinting. Recently, acoustic bioprinting and magnetic bioprinting have also been investigated for biomedical applications and there is still room to explore the practical applications of these new printing technologies.

#### 3D bioprinting technology

3D bioprinting products need to achieve the same level of complexity and detail as human tissue and organ structures, as well as multi-biological functionality. Moreover, the structure and function of different tissues and organs in the human body vary widely. Therefore, various 3D bioprinting technologies have emerged to meet the demand for printing high-precision complex structures that can simulate different tissues and organs. In this section we will elaborate on the main current 3D bioprinting technologies and their principles. And we also list their main advantages and disadvantages (Table 1).

**Table 1:** Advantages and disadvantages of mainstream bioprinting technology

Technology Type	Advantage	Disadvantage	Reference
Inkjet-based Bioprinting	Fast speed Low cost Strong simulation	Easy clogging of nozzles Limited ink viscosity Uneven ink size Poor sequence lines	[21, 23–26]
Laser-based Bioprinting	High resolution High cell viability Wide range of cell density Wide range of biomaterial viscosity	Photocrosslinker toxicity Photo-induced gene mutation Expensive Time consuming	[27–31]
Extrusion-based Bioprinting	Extensibility Easy to operate High versatility Low price Wide range of cell density Wide range of biomaterial viscosity	Low resolution Low cell viability	[12, 24, 32–36]

##### Inkjet (Droplet)-based bioprinting technology

**Fig. 1:**
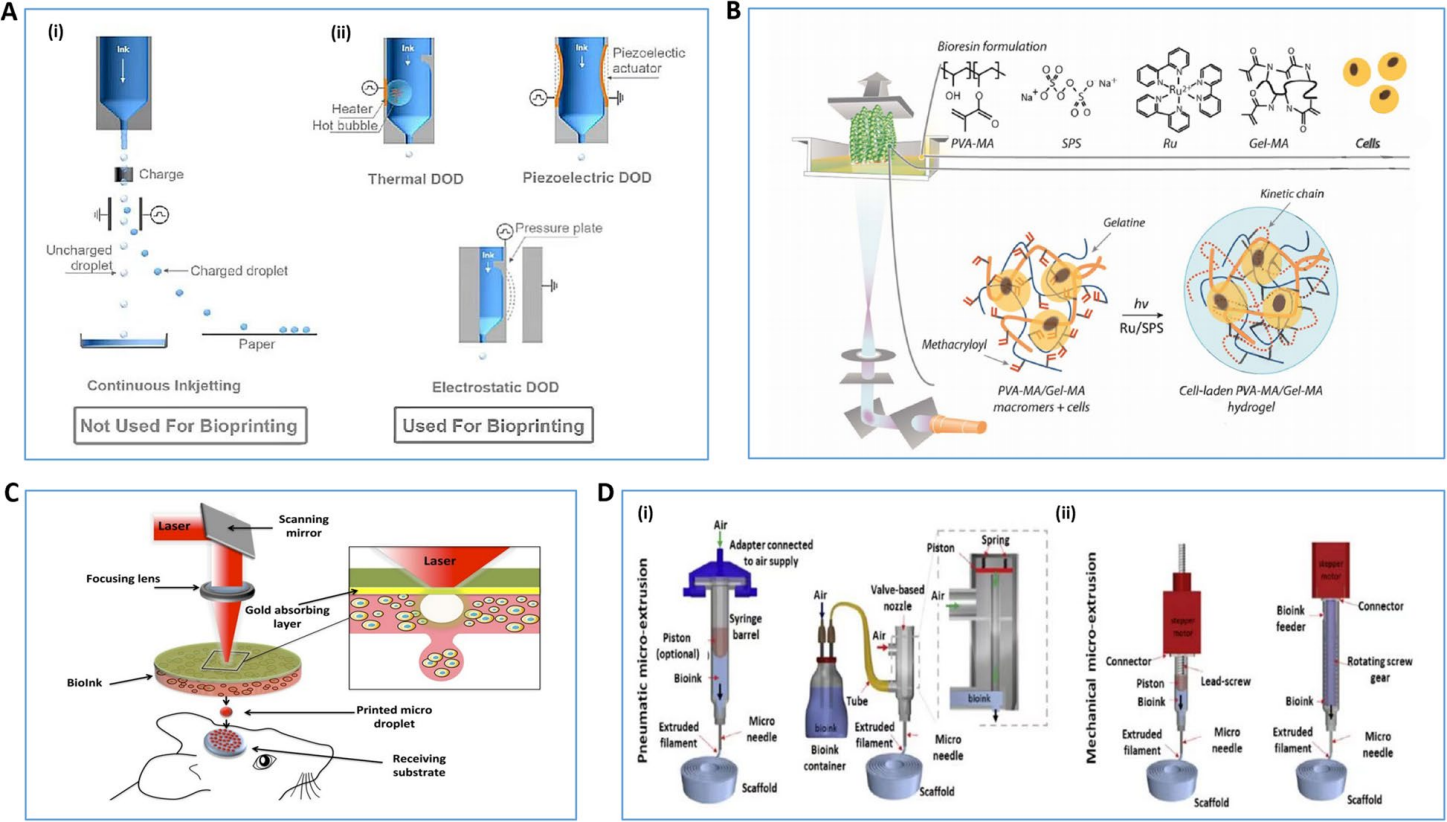
Schematic diagram of mainstream 3D bioprinting technology methods. **A** Schematic diagram of inkjet-based bioprinting method [37]. (i)The bioink is separated into continuous droplets, and the droplets are controlled by their own charge as well as by the peripheral electric field, (ii)Drip-on-demand inkjet printing designed with three drive methods: thermal, piezoelectric, and electrostatic can control the droplets to be ejected to any position to form a pattern. **B** Schematic diagram of the Stereo Lithography Appearance method [42]. **C** Schematic diagram of Laser-assisted Bioprinting based method [29]. **D** Schematic diagram of the extrusion-based bioprinting approach [35]. (i) mechanical force is generated by aerodynamic forces and (ii) mechanical force is generated by a piston or screw system. Reprinted with permission from Ref. [29, 35, 37, 42]

Inkjet-based bioprinting technology can print ink droplets in nano- and micron-scale volumes, depending on the printing needs. Also, since most of the current bioink is formulated with hydrogel material, this droplet-like printing method can ensure high resolution printing on a preset area [37] (Fig. 1A). Experiments usually use thermal or piezoelectric systems to briefly form the inkjet head to regulate the spraying of different sized droplets; they eject ink by mechanical energy generated by air pressure and current pulses, respectively [23, 38]. In addition, piezoelectric-driven bioprinters have higher cell bio-vitality than traditional thermal bioprinters that spray ink [39]. Inkjet-based bioprinting technology produces droplet sizes and print rates that depend on the fluid properties of the ink, the diameter of the nozzle, and the deformation frequency of the print head [24, 25, 32]. This technology also has the advantages of fast printing speed, relatively low cost, and strong simulation [32, 37]. However, it also has defects such as easy clogging of nozzles, limited ink viscosity, uneven ink size, and poor sequence lines [26, 40, 41].

##### Laser-based bioprinting technology

Laser-based bioprinting is a scaffold-free bioprinting technology. It mainly relies on laser source directional deposition or curing the printing material by means of optical cross-linking to build the structure of the printed product [43, 44]. Therefore, it can be mainly divided into: Stereo Lithography Appearance (SLA) and Laser-assisted Bioprinting (LAB). 1) SLA: This is a projection printing system that uses an ultraviolet or visiblelight projector to cure photosensitive ink into a specified area, and forms the desired model by layer-by-layer photopolymerization [42] (Fig. 1B). This technology eliminates the negative effects of shear stress on bioinks caused by nozzle printing technology, while enabling fast and highly accurate printing (resolution 5-300 µm) [27, 28]. 2) LAB: LAB uses laser pulses to deposit printing ink directly onto the collection substrate to build 3D biological models. The LAB system consists of three main components: a pulsed light source, a ribbon with a laser matrix containing a bioprinting material, and a collection substrate. The laser pulse is focused on the ribbon-induced absorption layer to form a localized evaporation producing a droplet containing the bioprinting material which falls towards the collection substrate and finalizes the print [29, 30]. These printing technologies are nozzle-free and have the technical feature of not directly touching the bioprinting material, eliminating damage to cells from shear stress. They guarantee high resolution and high cell viability printing and expand the range of cell densities for bioinks and viscosities of biomaterials (Fig. 1C). However, the cytotoxicity of photocrosslinkers and the mutation-inducing nature of ultraviolet (UV) light sources, as well as the high cost of building optical printing systems and the long printing process are still non-negligible drawbacks [29–31, 45, 46]. This greatly hinders the widespread adoption of laser-based bioprinting technology.

##### Extrusion-based bioprinting technology

Extrusion-based bioprinting prints biological materials by continuous extrusion using the mechanical force formed by pressure or distribution system (air, piston, etc.). Specifically, a bioink or biomaterial ink is placed in a disposable medical grade syringe. The ink is squeezed onto the sterile material by mechanical force generated by pressure or the dispensing system. During the printing process, the coordinated motion of the print head in coordination with the substrate in the three axes of space facilitates the high-precision deposition of multiple materials. This makes it possible to build large-scale complex 3D biological structures [33–35] (Fig. 1D). Extrusion-based bioprinting is preferred due to its scalability, ease of operation, and high versatility resulting from a widely applicable library of biomaterials [25, 26, 47]. In addition, compared to other technologies, extrusion-based bioprinting is able to deposit materials with a wide range of viscosities (30 mPa -s to over 6 × 10^7^ mPa ^−s^) and high cell densities at a relatively low cost with easy operation [14, 36, 48]. In contrast, the optimal resolution of this technology is low, reaching only 100 μm [49]. And the shear stress generated by extruding ink through tiny nozzles can affect cell survival and ultimately the entire product [50].

#### Emerging 3D bioprinting technologies

As research in the field of 3D bioprinting continues to intensify, higher demands are being placed on technologies including print scale, cell activity, and ink viscosity. To meet the higher demand researchers have developed several novel technologies and strategies.

Acoustic bioprinting technology is opening a new research avenue using single-cell manipulation techniques as well as surface acoustic wave technology to print 3D structures. In a relatively mild acoustic field environment, sound waves can move cells in different directions over a 3D space to produce complex 3D constructs. Also, as a non-contact printing technique, this method avoids nozzle clogging and damage to cell structure from pressure, heat, and shear stress [51]. In addition, magnetic bioprinting is also characterized by non-contact printing, direct endogenous synthesis of extracellular matrix (ECM) and high-precision spatial control, as well as the ability to rapidly print multiple tissue-like structures. Therefore, it is gradually being considered by researchers [52–54]. The main principle is that by pre-processing the bioink and exposing it to an external magnetic field, bioink can be magnetically guided [55, 56]. However, actual clinical studies of these technologies are relatively scarce. The potential of these technologies to combine multiple cell types and other biomaterials to print 3D biological structures should be explored.

**Fig. 2:**
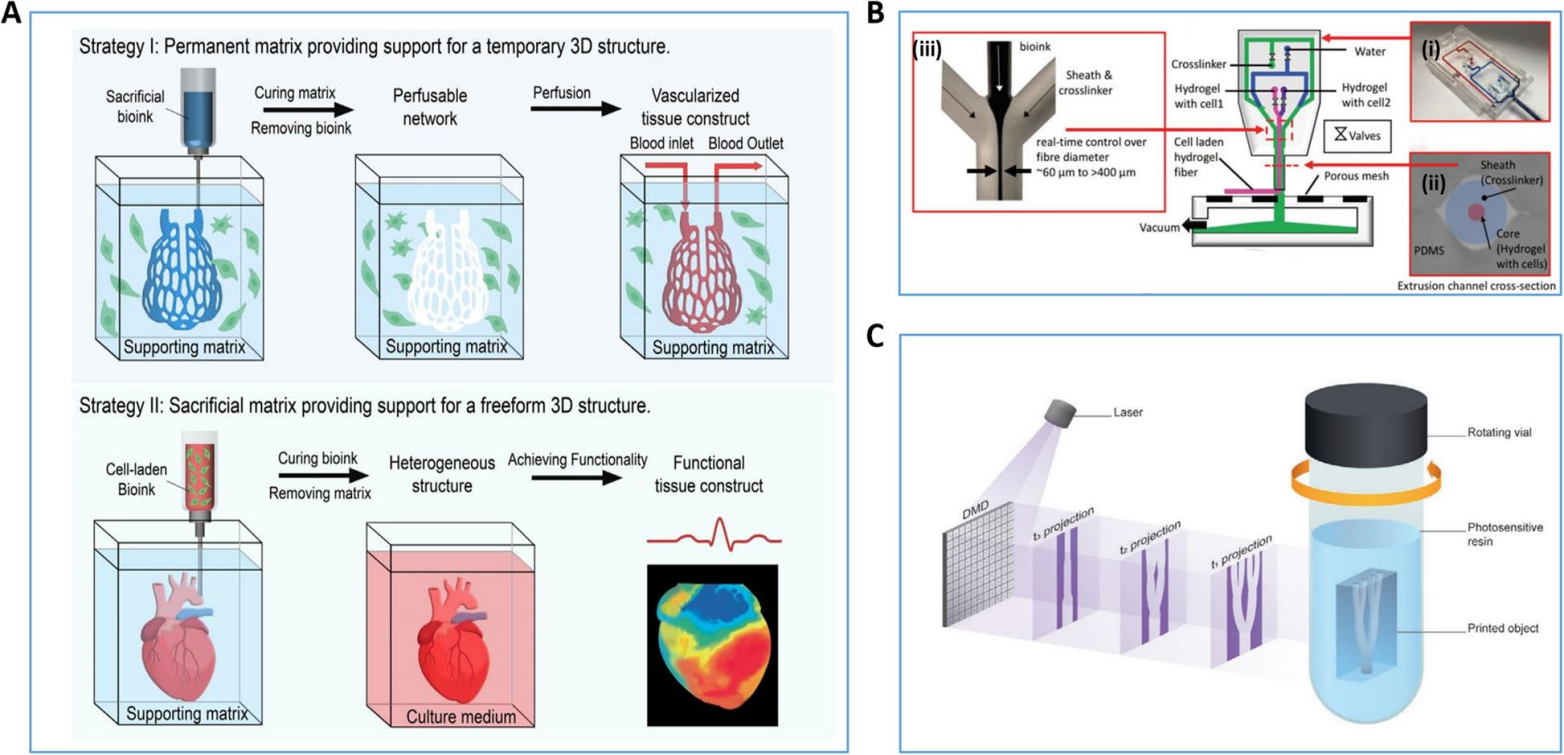
Schematic Diagram of New 3D Biological Printing Strategy. **A** Schematic diagram of the classical embedded bioprinting strategy [57]. **B** Schematic diagram of the microfluidic bioprinting strategy. (i) microfluidic print head with pneumatic valve, (ii) &(iii) coaxial flow focused extruder [59]. **C** Schematic diagram of the classical volumetric bioprinting strategy [62]. Reprinted with permission from Ref. [57, 59, 62]

In addition, to meet the demand for large-scale and high-precision bioprinting in research, researchers have proposed novel bioprinting strategies, such as embedded, microfluidic and volumetric bioprinting. In embedded bioprinting low-viscosity bioinks are extruded into a support tank, thereby increasing the structural complexity of the bioprinting tissue (Fig. 2A). This makes it possible to directly produce 3D volumetric biological structures. In microfluidic bioprinting by configuring a microfluidic system in an extrusion bioprinter, it is possible to create multi-component/multi-cellular biological tissue structures in a single print (Fig. 2B). And volumetric bioprinting can create a complete large living biological tissue structure in seconds, far exceeding the printing rates of traditional laser-based bioprinting technology with light projector systems (Fig. 2C). These new printing strategies are meeting the need for more demanding bioprinting products, and better mimic the structure and function of the patient's original tissue [57–62].

As discussed, both traditional and newer 3D bioprinting technologies, as well as the relatively new 4D bioprinting that will be mentioned below, have their own advantages and drawbacks. We need to recognize that there is no 3D bioprinting technology that is free of all defects, nor is there a printing technology that has all the advantages simultaneously. Therefore, in plastic surgery clinical and experimental research, for the actual clinical needs (e.g., skin wound healing, rhinoplasty, ear reconstruction, etc.), it is necessary to select the right bioprinting materials, pick or combine different bioprinting technologies, in order to finally develop a suitable 3D bioprinting strategy.

### Biomaterials for 3D bioprinting

The application of 3D bioprinting technology often involves multiple areas of tissue engineering (such as skin, bone, cartilage, etc.). Moreover, the ECM of different tissues has different properties, and the cellular structure within different tissues varies. Therefore, developing a universal printing material is not realistic. Then it becomes crucial to choose the right printing material to fit the different organ tissues. The selection of printing materials is mainly based on the characteristics of printability, biocompatibility, and mechanical properties of bioprinting materials. 1) Printability refers to the moldability of the bioprinting material, which includes a tunable material viscosity, the ability to rapidly transition the material from the sol to the gel state, and a wide range of printing parameters. 2) Biocompatibility refers to the biomimetic ability of bioprinting materials. As cells need to grow, add value, and differentiate in an environment where bioprinting materials are present, bioprinting materials should be able to mimic the biological environment of the repair site as closely as possible. Some printing products need to be retained in the body for a long time, requiring low cytotoxicity. 3) Mechanical properties refer to the requirement that the bioprinting material has a certain structural strength to ensure that the subsequent culture and implantation process will not be structurally deformed. Moreover, some bioprinting constructs may undergo nutrient perfusion as well as biological degradation during in vitro culture. The lack of certain mechanical properties will eventually lead to the destruction of the structure of the bioprinting product. In summary, the selection and design of printing materials requires consideration of factors such as bioprinting technology, structural requirements, and the type and growth of cells. A reasonable combination of these three properties is assembled to finally arrive at the optimal choice [63–66]. Next, we will introduce the 3D bioprinting materials commonly used today and briefly describe their respective properties (Table 2).

**Table 2:** Characteristics of bioprinting materials

Type of material	Materials	Basic Performance	Special Performance	Reference
**Inorganic Biomaterials**	Metals such as titanium and its alloys	High strength; Low modulus of elasticity; Low density	/	[61, 62]
	Bioceramic materials	Biocompatibility; Osteoconductivity; Corrosion resistance; High compressive strength; Low tensile strength	Potential for long-term bone tissue implants	[63, 64]
	Clay, hydroxyapatite, graphene, carbon nanotubes, etc	Mechanical Properties; Printability	Biomineralization	[65, 66]
**Synthetic Polymers**	PCL	Biocompatibility; Low biodegradation rate	Osteogenesis (compare with PLA)	[67, 68]
	PLA	Good ductility; Good stiffness; Machinability; Biocompatible; Fast biodegradation rate	/	[69, 70]
	PU	Biocompatibility; High elasticity	Adjustable physicochemical properties and degradation rates	[71]
**Natural Biopolymers**	Alg	Low cost; Biocompatibility; Tunable rheological and mechanical properties	Adjusting the concentration can change the cell survival rate	[72–75]
	COL	Easy Extraction Printability; Biocompatible	Mixing and cross-linking other biomaterials to modulate biological functions and mechanical properties	[76–80]
	GEL	Biocompatibility; High water absorption; Biodegradability; Non-immune; Thermal responsiveness RGD base sequence	Photosensitive materials prepared by methacrylating modification can be used for light-curing printing	[81–84]
	HA	Biocompatibility; Biodegradability	Differences in the mechanical and biological properties of hydrogels prepared from HA with different molecular weights	[85, 86]
	SF	Biocompatibility; Biodegradability; Processability; Good mechanical properties	β-sheet stacking structure, low viscosity and other characteristics hinder its application	[87–90]
	CHO	Biocompatibility; Biodegradability; Antibacterial properties	Demonstrates healing-promoting ability in chronic wounds	[91, 92]
	dECM	Biocompatibility; Provides a cell-specific microenvironment; Preserves some cell-specific functions	Compensate for the lack of mechanical and biological properties by dECM modification	[93–95]
**Composites**	PEG diacrylate + GelMA	Biocompatibility; Good mechanical properties; High resistance to degradation	High fidelity and tunable mechanical properties	[96]
	CHO + COL	Biocompatibility Printability; Good mechanical properties	Adjust the mechanical properties and printability of bioprinting products by changing the gelation temperature	[97]
	PLA + PCL	Good mechanical properties; Biodegradability	Poor biocompatibility is still a difficult problem to solve	[98, 99]
	HAp + GEL	Biocompatibility; Good mechanical properties	Excellent shape fidelity; mechanical strength comparable to that of native bone; and enhanced bioactivity in terms of cell proliferation, attachment, and osteogenic differentiation	[100]

#### Inorganic biomaterial

Inorganic materials, mainly metals and bioceramics, have been widely used in the biomedical field. Metallic biomaterials such as titanium and its alloys are widely used in bone tissue engineering because of their high strength, low modulus of elasticity and low-density structure. For example, the two teams of Wang and Xu successfully prepared different bone tissue bioscaffolds using Ni46.5Ti44.5Nb9 and Ti35Zr28Nb alloys, respectively. The scaffolds showed excellent mechanical property. Moreover, in vitro experiments showed that the cells attached to the scaffolds grew and proliferated well [67, 68]. In addition, bioceramic materials have good biocompatibility, osteoconductivity and corrosion resistance. And, although it does not perform well in terms of tensile strength, its performance in terms of compressive strength is high (data show that its compressive strength is ten times higher than its tensile strength). Making it a very promising material for 3D bioprinting [69]. In particular, the calcium phosphate composition of bioceramics is similar to the mineralogical structure of natural bone. Therefore, it is considered to have potential as a long-term implant for bone tissue [70]. Further, inorganic materials, such as clay, hydroxyapatite, graphene, carbon nanotubes, and other silicate nanoparticles are also being used in 3D bioprinting research due to their respective mechanical properties, printability, and other characteristics [71]. It should be noted that most inorganic materials do not exhibit extremely high biocompatibility, so they are more often designed and formulated as biomaterial inks for printing 3D scaffolds in practical applications. However, some inorganic materials can also be mixed in hydrogels when designing biomineralization strategies for bioinks, adjusting the microenvironment to induce targeted cellular functions. For example, Neufurth et al. have designed a polyphosphate(polyP)-rich bioink composed of N, O-carboxymethyl chitosan, alginate and gelatin, and polyP which improves the survival and migration propensity of mesenchymal stem cells (MSCs). Moreover, it promotes the differentiation of MSCs to mineral-depositing osteoblasts [72]. This type of research expands the scope of application of inorganic materials in bioprinting.

#### Synthetic polymers

Currently, a wide range of synthetic polymers can be used in the preparation of biomaterial inks. However, synthetic polymers usually have good mechanical properties but lack robust biocompatibility. Thus, among synthetic polymers, the most widely used are still biomaterials represented by polycaprolactone (PCL), polylactide (PLA) and polyurethane (PU).

PCL shows good biocompatibility and low biodegradation rate, and is now widely used in biomedical fields. For example, Kolan's team added a highly angiogenic borate bioactive glass to PCL for use in bioink, validating the feasibility of a bioink with a PCL/bioglass component. Moreover, the osteogenic effect of PCL-based 3D scaffolds is significantly better than that of PLA-based 3D scaffolds. However, the hydrophobic nature of PCL often leads to a lower cell survival rate of the constructed bio-scaffolds [73, 74]. PLA has good ductility and stiffness, processability, biocompatibility, and a fast biodegradation rate. Sun et al. designed two stereoisomers based on PLA, [poly-l-lactic acid/polyethylene glycol/poly-l-lactic acid] and [poly-d,l-lactic acid/polyethylene glycol/poly-d,l-lactic acid] which changed the hardness of their constituent hydrogels and further broadened the application of PLA. However, the release of acidic by-products during degradation and the brittleness of PLA, limit its application in tissue engineering [75, 76]. PU has become one of the most popular synthetic polymers in the biomedical field due to its biocompatibility, high elasticity, adjustable physicochemical and degradation rate properties [77]. Although these synthetic polymers have been studied extensively in the field of bone and cartilage tissue engineering, synthetic polymers do not equal the biocompatibility of natural polymers. Therefore, synthetic polymers play a role in 3D bioprinting mainly as a physical and mechanical framework support. They can be prepared in combination with growth factors and other components to become biomaterial ink involved in 3D scaffolds, bioimplants, and other 3D printed constructs without cellular components.

#### Natural biopolymer

There is a wide range of natural biopolymers, some are water-soluble. This means that these natural polymers can be cell-friendly biosolvents and can be prepared as hydrogels. Because of their mobility, they can all theoretically be designed as bioinks together with seed cells. Then, with computer aided design models, they can be 3D bioprinting according to the principle of layer-by-layer printing. These hydrogels not only exhibit viscoelastic behavior that mitigates shear-induced cell damage during printing, but also mimic the complex microenvironment of the natural extracellular matrix (ECM). In past research, many natural hydrogels (e.g., Alginic acid, gelatin, collagen, fibrin, and decellularized ECM [dECM]) have been considered ideal materials for the preparation of bioinks due to their biocompatibility, intrinsic bioactivity, and structural similarity to natural ECM).

Alginic acid (Alg), also known as fucoidan, is an anionic polysaccharide extracted from brown algae. Due to its relatively low cost, good biocompatibility, easily adjustable rheological and mechanical properties, as well as the ability to be chemically cross-linked by divalent cations (e.g., Ca^2+^, Sr^2+^ and Ba^2+^), it is widely used in the preparation of bioinks. However, Alg-based hydrogels often have insufficient viscosity, resulting in reduced cell viability [78–80]. Park's team showed that the viscosity of cell-containing sodium Alg hydrogels is highly dependent on factors such as polymer concentration, molecular weight, as well as cell phenotype and density. Usually, when cells are mixed into sodium Alg hydrogels with high polymer concentrations, their biological activity is greatly limited after chemical cross-linking. The lower concentration of sodium Alg hydrogel helps improve cell viability and proliferation [81]. However, if the concentration of sodium Alg hydrogel is consistently reduced, even after chemical cross-linking, the mechanical strength of the 3D structure of the final printed product will be drastically reduced, leading to the failure of the print.

Collagen (COL) is a protein that is widely found in human connective tissue and ECM. Due to its ease of extraction, printability and biocompatibility, COL has been used in the bioprinting of various organ tissues. In particular, type I and type II COL have been widely used for bone and cartilage repair [82, 83]. Moreover, researchers have indicated that the biofunctionality and printability of COL-based hydrogels can be enhanced by mixing or cross-linking with other bioprinting materials (agarose, GEL, fibrin, calcium phosphate, etc.) [60, 84–86]. However, other biological properties of COL-based hydrogels, such as cytocompatibility, will inevitably be affected when other printing materials are added. Thus, selecting a properly designed bioink formulation is key to the success of this type of hydrogel printing.

Gelatin (GEL), a derivative of COL, is a protein with low cytotoxicity and water solubility, which can be extracted from a variety of mammals. Although GEL and its derivatives are less viscous, they have been widely used in 3D bioprinting due to their excellent biocompatibility, high water absorption, rapid biodegradability, low immunogenicity, thermal responsiveness, and the presence of Arginine-Glycine-Aspartic Acid (RGD) motifs [87, 88]. Moreover, GEL can be prepared into a photosensitive hydrogel by methacrylating modification (GelMA), that can be excited by UV or visible light for photocuring reactions. This type of hydrogel has excellent biocompatibility along with better printability and mechanical properties. However, the UV cross-linking may damage cellular DNA [89, 90]. A more secure and reasonable printing method is something that research scholars should explore further.

Hyaluronic acid (HA) is a non-sulfated glycosaminoglycan composed of D-glucuronic acid and N-acetyl-D-glucosamine, which is the main component of ECM. HA has good biocompatibility and biodegradability and plays an important role in cell proliferation, angiogenesis, and cell receptor interactions [91, 92, 101]. It was found that the mechanical and biological properties of hydrogels varied when prepared using HA with different molecular weights. Controlling the molecular weight of HA to prepare suitably functional hydrogels helps improve bioprinting [93].

Silk fibroin (SF) is a natural polymeric protein extracted from natural silk. With its excellent biocompatibility, biodegradability, processability, and excellent mechanical properties, it is considered by researchers to be a promising bioprinting material [94, 95, 102]. However, its β-sheet stacking structure, low viscosity and other properties lead to difficulties in 3D bioprinting applications [103, 104]. Nevertheless, Kim et al. used methylation modification to enhance the rheological properties of SF hydrogels, which allowed for the preparation of printed products with good biocompatibility and mechanical properties [105]. Exploring further modifications to enhance the printability and mechanical properties of SF shows excellent research prospects.

Chitosan (CHO) is a natural polysaccharide formed by deacetylation of chitin extracted from shrimp shells. Due to its good biocompatibility, biodegradability, and antibacterial characteristics, CHO is widely used in the preparation of bioprinting products such as biological scaffolds and drug delivery systems [106]. Moreover, CHO-based biologic constructs have shown excellent healing promotion in chronic wounds [107]. Bioink based on CHO research design is expected to manage the healing of chronic wounds such as clinical diabetic ulcers.

In recent years, the concept of dECM in 3D bioprinting has gradually emerged. Specifically, dECM is the removal of the original cells from the target tissues and organs, while preserving the ECM fraction. After cell removal, the remaining constituents of the target tissue are highly preserved. Moreover, the formulation of these components as bioink provides an excellent cell-specific microenvironment which preserves cell-specific functions [108, 109]. The human body has different tissues and organs, and it is difficult to realistically simulate the microenvironment of human cells using only one, or several combinations of, designed bioprinting materials. And the dECM-based design of bioink is expected to solve this problem in a real sense. Currently, dECM has been applied to skin, cartilage, fat, and other tissue engineering applications. Although dECM still has problems such as compositional inconsistency, low mechanical properties, and potential immunogenicity [108–110], researchers are gradually compensating for its mechanical and biological deficiencies through modification [111]. There is no doubt that the dECM-based bioink is a transformative breakthrough in 3D bioprinting technology.

It is worth noting that the bioink based on most of the above materials has poor mechanical properties of the structure after printing, making it difficult to meet the needs of practical applications. Currently, many experiments have shown that the printed structure can undergo thermal, chemical, and light curing to improve the structural stability of the product. In addition, light curing technology is the most widely used, because it can directly improve the mechanical properties of biologic structures while meeting the requirements of cytocompatibility, degradability, and ease of operation [112]. Specifically, this involves the use of photosensitive hydrogels as one of the components of bioink. After printing the bioink into a 3D biological construct, it is exposed to artificial UV or visible light. UV cross-linking of the photosensitive components in the printed structure, leading to curing of the printed structure. Both light duration and temperature can affect the mechanical properties of the final printed structure [112, 113]. In addition to the GEL mentioned above which can be prepared as GelMA, such as HA, SF, algae gum, PCL, etc. can be prepared as corresponding photosensitive biomaterials by methacrylating. These photosensitive materials generated by methacrylating modifications have enhanced mechanical properties through UV cross-linking reactions while retaining the biological properties of the underlying materials, and greatly expanding the scope of applications of 3D bioprinting in the field of regenerative medicine [114–117].

As mentioned at the beginning of this section, there is still no one universal 3D bioprinting material. However, further expansion of the range of applications for different 3D bioprinting materials is indeed one of the goals that a wide range of researchers are currently striving to achieve. Fortunately, the advent of composite technology has allowed various bioprinting materials to exploit their own strengths while compensating for their respective shortcomings in terms of mechanical properties and biological characteristics [118]. Firstly, the further use of natural polymeric materials for 3D bioprinting is hindered by their often lack of good mechanical properties and printability. Moreover, synthetic polymers have better mechanical properties but are less biocompatible. Therefore, a bioink strategy combining both natural biomaterials and synthetic polymers is a more likely solution to be considered. For example, García-Lizarribar et al. combined poly (ethylene glycol) diacrylate with GelMA to obtain a photopolymerisable hydrogel mixture. The bioink prepared based on this composite hydrogel prints 3D structures with better mechanical properties and resistance to degradation, which can be applied to 3D bioprinting of muscle tissue [96]. Suo et al. have designed a composite bioink based on chitosan and collagen. They have greatly enhanced the mechanical properties and printability of the chitosan/collagen composite bioink through hydrogen bonding [97]. Similarly, composites based on different types of synthetic polymers can also be used for 3D bioprinting(e.g., combination of PLA and PCL) [98]. However, bioinks designed solely based on synthetic polymers can lead to relatively poor biocompatibility of the final printed product, which remains a problematic issue [99]. In addition, as 3D bone tissue scaffolds require good biocompatibility and high strength mechanical properties. Single types of bioprinting materials are difficult to meet these needs, so composite based 3D bone tissue scaffolds have been a hot topic of research [119–121]. In particular, the combination of inorganic materials and natural biomaterials provides excellent mechanical properties while mimicking the microenvironment of bone tissue cells to the greatest extent possible (e.g., the combination of Hydroxapatite and GEL) [100, 122]. In short, although different bioprinting materials have their own strengths and weaknesses, we can highlight the strengths and compensate for the weaknesses of the materials as much as possible by combining different bioink strategies. With this approach, we can more easily broaden the range of applications of different bioprinting materials, design appropriate bioink strategies for different clinical needs and accelerate the clinicalization of 3D bioprinting.

#### Functionalization of bioink

In addition to bioprinting materials, the components of bioink require some functionalized formulations (e.g., extracellular vesicles, growth factors, seed cells, etc.) to refine their biological properties and enable them to perform specific biological functions to meet the needs of different practical applications. Extracellular vesicles (EVs) are cell-secreted nanoscale vesicles that can be extracted from the extracellular fluid (e.g., blood, urine, milk, etc.) from a wide variety of organisms. In particular, the paracrine action of MSCs-derived EVs (MSCs-EVs) is thought to have a function in promoting repair and regeneration [123, 124]. It is well recognized by researchers that MSCs-EVs stimulate high expression of target cell-related signaling pathways and induce tissue repair and regeneration mainly through their encapsulated proteins, RNA, lipids, and other components [125]. The design of EVs as functional formulations to enhance the biological properties of bioinks is a current research hotspot and has been applied to the construction of different 3D biological constructs [126–128]. The growth factor (GF) component of bioink also plays an important biological function, regulating the microenvironment to be suitable for cell growth and differentiation [129]. Selecting a suitable GF for bioink can specifically and rapidly promote the growth and differentiation of target cells, and enhance the cell purity within the bioink. Therefore, GFs have been widely used in the bioprinting of various tissues and organs [130–133]. However, too rapid release of GFs is not conducive to cell growth and differentiation. The slow-release strategy designed by Yi et al. promoted better performance of GF’s biological functions [134], expanding the application of GF in 3D bioprinting.

The seed cells encapsulated in bioink are a key part of bioprinting. Moreover, MSCs are the preferred seed cells due to their abundant source, multidirectional differentiation ability, low immunogenicity, and paracrine effect [135]. Due to the ease of extraction and low cost, human umbilical vein endothelial cells with stem cell potential, as well as MSCs such as human pulp stem cells, fat-derived MSCs, and bone marrow MSCs, have been widely used in 3D bioprinting [135–137]. In addition, mature cells derived by stem cell-derived differentiation or extracted from primary, mature, human tissue, can also be used as seed cells of choice. Because the structure and function of mature cells are fixed, printed 3D biological structures can play a more stable role in specific tissues and organs [138, 139].

However, just as it is difficult to have a perfect bioprinting technology, even with the emergence of dECM-based biomaterial applications, there is still no perfect bioprinting material. Overall, inorganic biomaterials generally exhibit excellent mechanical properties but are not ideal in terms of biocompatibility and printability. Synthetic polymers generally have good mechanical properties and printability, but are not as biocompatible. Although natural biopolymers are mostly biocompatible, they usually have poor mechanical properties. Moreover, the biological characteristics of bioink without functional components are not optimal, but the purely biofunctional components are separated from the bioink environment and will be rapidly degraded. No matter the advantages and disadvantages of the bioprinting material, it is not advisable to use only a single bioprinting material to produce printing products. Combinations of two or even more biomaterials allow for design of biomaterial inks or bioinks to compensate for each other's material defects. This allows us to better meet the need for extracellular environment simulation and ensure the structural properties of the printed product.

### The fourth dimension of bioprinting—4D bioprinting

4D printing is essentially a 3D printing technology. 4D printing is characterized by a multi-material printing capability over time, or a customized material system that can change from one shape to another. Therefore, 4D bioprinting technology can be summarized as follows: special 3D bio-constructs with biological activity are exposed to a predetermined stimulus, and their function, shape, and properties can change over time [26, 140, 141].

#### 4D Bioprinting based on shape transformation

Most common in 4D bioprinting are bioprinting products capable of shape transformation. It is mainly divided into two categories: restoring and not restoring the original shape after transformation [141]. Researchers have now discovered multiple ways to alter the original structure of the printed product, including manual folding, cellular traction, and stimulus response. However, it is difficult to achieve precise control using manual and cell traction folding of the 4D printing product structure. Therefore, in order to achieve refined control, smart printing materials based on stimulus response are still the most widely used [142]. Stimulus-responsive materials can undergo conformational changes in response to specific stimulus conditions (e.g., temperature, pH, humidity, electricity, magnetic field, light, acoustics, or a combination of these stimuli). These conditions can be broadly classified as physical and chemical stimulation.

##### Smart Printing materials that respond to physical stimulation

**Fig. 3:**
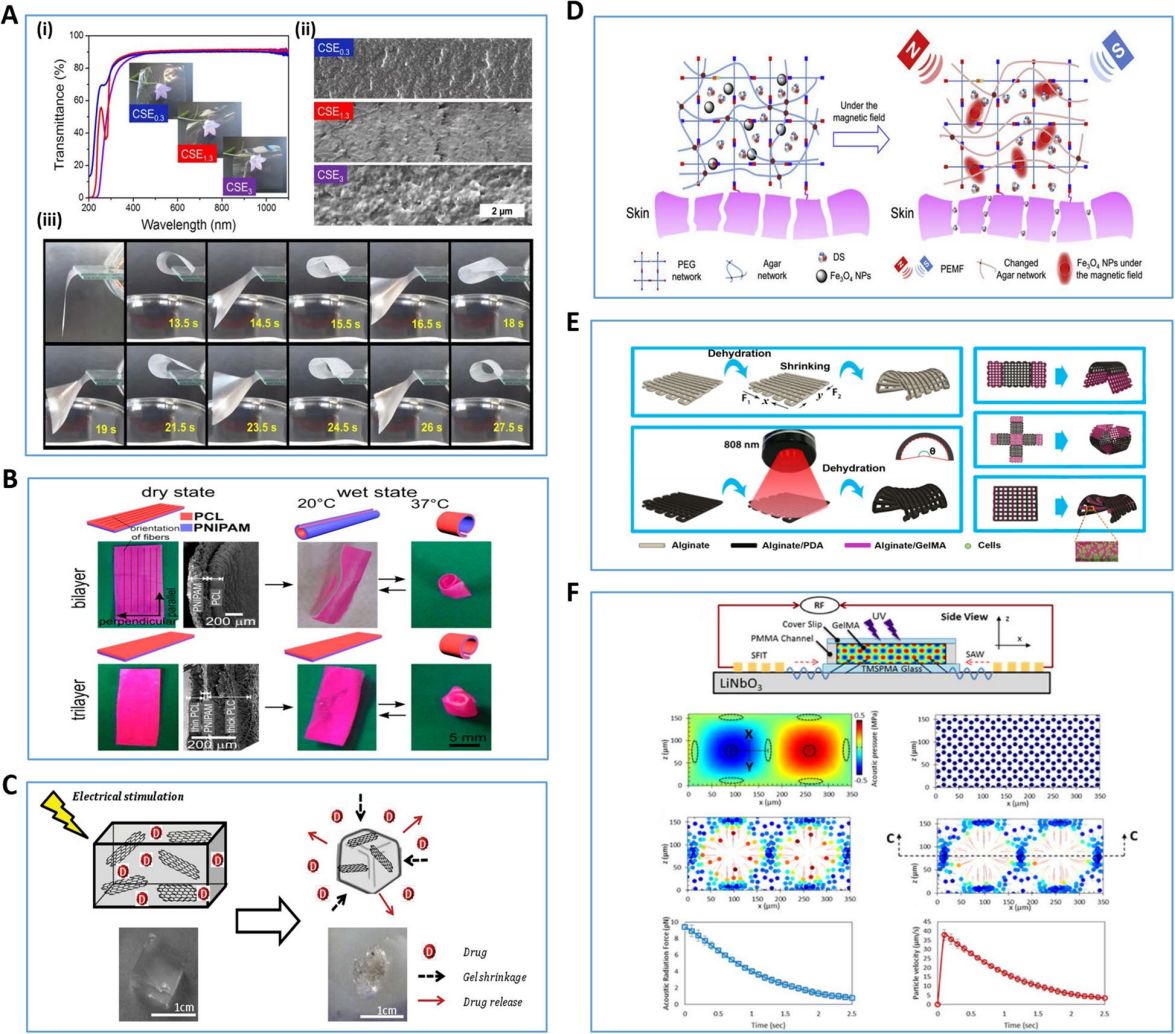
Various types of Smart Printing Materials that Respond to Physical Stimulation. **A** Schematic diagram of a moisture-responsive 4D bioprinting product [143]. (i) transparency capability demonstration, (ii) scanning electron microscope image, (iii) printed product affected by moisture beneath it, which in turn transforms the morphology. **B** Schematic diagram of temperature-responsive 4D bioprinting products [144]. **C** Schematic diagram of a 4D bioprinting product with electrical stimulation response [146]. **D** Schematic diagram of a magnetically responsive 4D bioprinting product for synergistic treatment of soft tissue injury system [147]. **E** Schematic diagram of a photoresponsive 4D bioprinting product [148]. **F** Schematic diagram of sound-responsive 4D bioprinting products. Numerical modeling demonstration of the displacement profile generated in GelMA prepolymer solution [149]. Reprinted with permission from Ref. [143, 144, 146–149]

Cellulose stearate-based bilayer smart biomaterials can change their shape by shrinking or expanding in response to changes in humidity. And it enables fast reversible bending motion and continuous shape transformation (Fig. 3A). However, it is important to note that wet-sensitive 4D printing materials have only appeared in the early studies of 4D bioprinting. Although they can be used for 4D bioprinting, the cells in the bioink need to maintain constant humidity and osmotic pressure during the culture process, so morphological transformation of biomaterials is limited [143]. In addition, in response to temperature stimulation, researchers have developed and designed smart materials that can respond to temperature (Fig. 3B). Currently, temperature-responsive materials designed with a base of poly(N-isopropylacrylamide) are considered to be among the most promising materials for 4D bioprinting. Apste et al. has developed a 3D scaffold that is capable of self-curling in a 37 °C water environment [144]. However, due to limitations such as low biocompatibility, hydrophobicity, and non-degradability [145], the practical application of temperature-responsive smart materials in bioprinting has yet to be explored further.

With better biocompatibility and mechanical properties, smart materials that respond to electrical stimulation are rapidly developing in the biomedical field [150]. Smart materials that respond to electrical stimulation achieve shape transformation of materials in two main ways: by using electroactive materials and by using cellular structures driven by electrical stimulation to achieve movement. 1) Electroactive materials: Under electrical stimulation, polyelectrolyte polymers can swell, shrink, fold or bend, and these shape shifts can be regulated depending on the direction and strength of the electric field. Currently, conductive polymers with good biocompatibility, such as polypyrrole, polyaniline and polythiophene, are being widely used in hydrogel research, showing good potential for 4D bioprinting [151, 152]. In addition, in recent years, electro-responsive materials based on carbon-based nano-biomaterials such as graphene and carbon nanotubes have been employed in 4D bioprinting. Servant's team developed a macroporous scaffold based on graphene with good mechanical properties, the ability to respond to electric fields and thermal properties. It also exhibited the ability to promote neurogenic differentiation of human bone marrow MSCs [146] 2) Cell-driven by electrical stimulation: refers to the remote control of cells to a predetermined location or directing cells to a specific direction by the action of an electric field [153]. The feasibility of the above design was demonstrated by culturing skeletal muscle strips on a 3D structure based on polyethylene glycol bisacrylate hydrogels [154] (Fig. 3C). However, during the use of electrically responsive smart material technology, as the applied current increases, local overheating as well as cell rupture and death may occur, which will cause the final printing product to fail. The design of safer electrically stimulated smart materials may be a hot topic of research in future studies.

Researchers have designed smart materials that respond to magnetic fields using magnetic particles and nanoparticles (e.g., containing magnetic components such as iron, cobalt, nickel and their oxides) (Fig. 3D). Several studies have shown that these materials can be used to design drug release systems. For example, by combining Fe_3_O_4_ nanoparticles with polyethylene glycol agar hydrogels, drug delivery systems can respond to magnetic field stimulation [147]. Moreover, due to the paramagnetic properties of nanoparticles, magnetic levitation assembly of cells or micro tissues is realized [155]. However, smaller nanoparticles (< 50 nm in diameter) can cross biological membranes and adversely affect tissue function by inducing inflammation, generating reactive oxygen species, impeding DNA function, and inducing apoptosis. Therefore, we should focus on the biocompatibility of their nanoparticle components when selecting smart materials that respond to magnetic field stimulation [156].

As the selection of 4D bioprinting materials continues to grow, researchers have developed smart biomaterials that can respond to optical and acoustic stimulation. In particular, photosensitive materials exhibit high strain shape memory and self-healing capabilities by virtue of their polymer chain photoisomerization and photodegradation response mechanisms (Fig. 3E). Therefore, it has been widely used in tissue engineering and biomedical fields [148, 157]. However, the phototoxicity of photoinducers and the attenuation effect of light penetration through tissues still limit this technique, which is difficult to overcome. In recent years, acoustically sensitive materials have received much attention due to their non-contact stimulation and their fast and accurate shape transformation capabilities (Fig. 3F). However, this material is currently limited to only linear modes of transformation of the material shape, and only homogeneous-cell-populations can be constructed [145, 149]. Research on the use of this material to construct complex heterogeneous constructs should be further explored.

##### Smart printing materials that respond to chemical stimulation

**Fig. 4:**
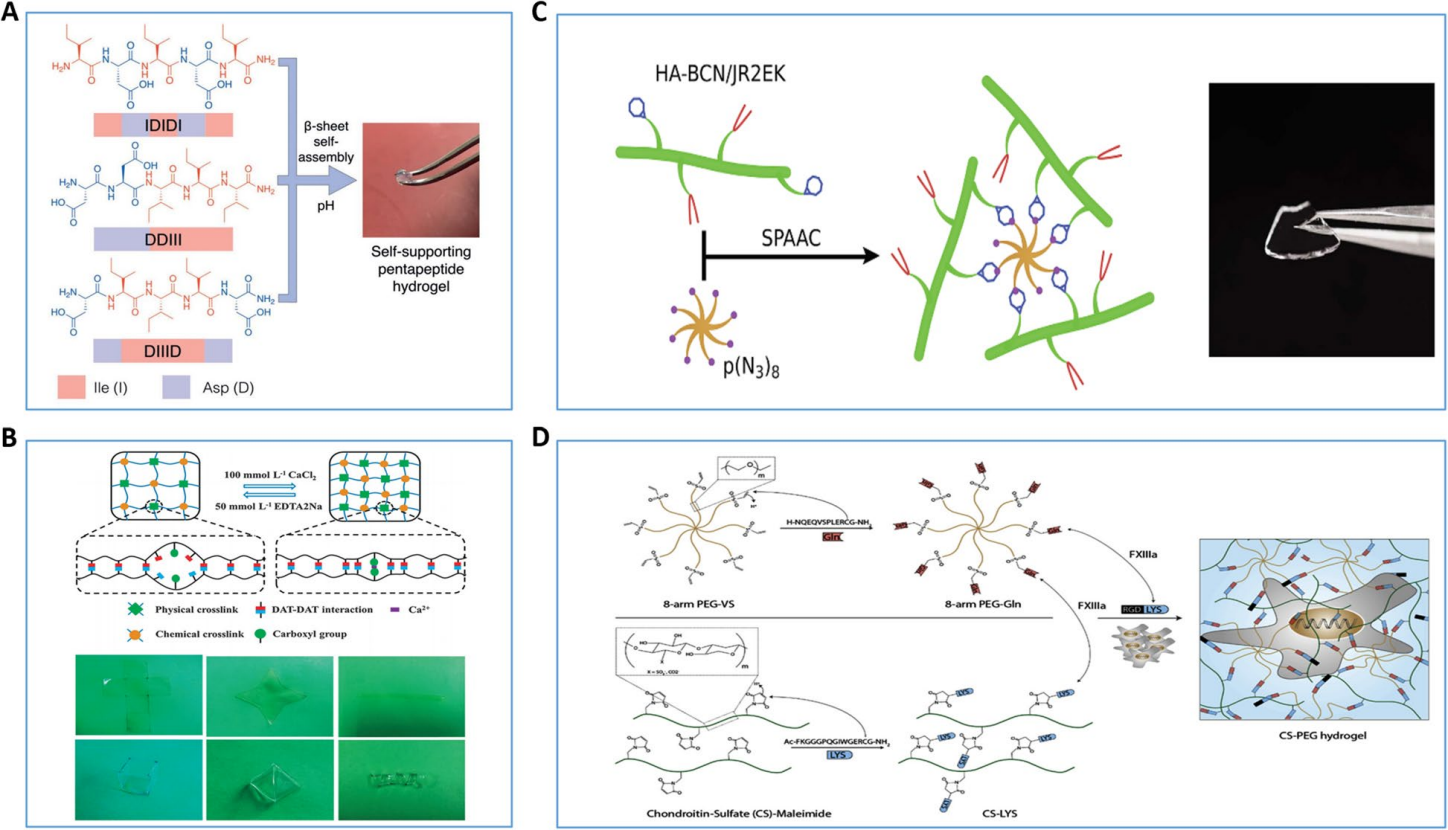
Various types of Smart Printing Materials that Respond to Chemical Stimulation. **A** Schematic diagram of pH-responsive 4D bioprinting products [158]. After pH stimulation, the function of the hydrogel is transformed. **B** Schematic diagram of ion-responsive 4D bioprinting products. Ca.^2+^ triggers shape transformation of PVDT-PAA-PBS(PVDT-PAA is synthesized by 2-vinyl-4,6-diamino-1,3,5-triazine, acrylic acid, and polyethylene glycol diacrylate; PBS is phosphate buffered saline) hydrogels into box-like, pyramid-like, and spring-like shapes [161]. **C** Schematic diagram of 4D printed products based on bio-orthogonal crosslinking design [162]. **D** Schematic diagram of a biologically responsive 4D bioprinting products [163]. CS-PEG (an enzymatically formed chondroitin sulfate and poly (ethylene glycol) based hybrid hydrogel system) hydrogel formation mediated by transglutaminase XIII factor. Reprinted with permission from Ref. [158, 161–163]

The pH of the environment in which the material is placed is controlled by adding polyelectrolytes containing weakly acidic or basic groups (such as carboxyl, pyridine, sulfonic acid, phosphate, etc.) to the printing material. These groups then release or accept protons, which cause structure or property shifts in this pH-responsive material (Fig. 4A). The components of pH-responsive smart materials are divided into two categories: basic and acidic monomeric polymers. Basic monomer polymers behave as cationic polymers under acidic conditions, while acidic monomer polymers behave as anionic polymers under basic conditions [156, 158]. However, because of their poor mechanical properties, it is often necessary to add other synthetic materials when designing this smart material to ensure the structural stability of the final product [159, 160]. Moreover, these PH-responsive materials inevitably produce compounds when they change shape under the influence of acid–base environment. The potential cytotoxicity of these chemistry by-products and their possible impact on the structure of the material need to be avoided during the design process [140].

In addition, in order to make materials with higher structural strength, some materials containing multivalent ions (Ca^2+^ or Zn^2+^) can be designed as smart materials in response to ionic stimulation. For example, hydrogels designed based on hydrogen bonding-calcium ion interactions exhibit shape memory capabilities under reversible calcium ion action. Moreover, these materials showed good biocompatibility and biodegradability in experiments [161, 164, 165] (Fig. 4B). However, multivalent ions such as Ca^2+^ or Zn^2+^ regulate vital bodily functions and are important indicators for monitoring many diseases. Therefore, the inclusion of a dynamic monitoring and response system for polyvalent ion concentration in this smart material design to avoid the impact of polyvalent ions in the material on clinical data should be explored in the future.

In addition to the above-mentioned smart materials based on ionic cross-linking, researchers have designed biostimulus-responsive materials using the feedback regulation of biomolecules (nucleic acids, proteins, peptides, etc.) in the human body. They function through adjustable bio-covalent orthogonal cross-linking and specific peptide folding-mediated effects to achieve dynamically regulated cross-linking and functionalization of printed biomaterials (Fig. 4C). This technology has been used to create an ECM environment that simulates dynamic changes and to design a modular peptide system capable of dynamically changing 3D printed structures [162, 166]. As highly specific and functional biomolecules, enzymes play a key role in regulating physiological functions. Biostimulus-responsive smart materials designed based on bioenzyme stimulation have great research potential. Experiments have already demonstrated the feasibility of using hydrogel materials loaded with multiple enzymes for bioprinting applications [163, 167] (Fig. 4D). However, in recent years, the research on 4D printing materials of biological enzymes has been mostly limited to the application of the degradation function of enzymes, and smart materials designed based on stimulation of other functions of biological enzymes need to be further explored.

#### 4D Bioprinting based on functional transformation

4D bioprinting for functional transformation is a concept that has been gradually refined. Unlike 4D bioprinting based on shape transformation, this printing technology is currently considered primarily for the printing and differentiation of stem cells to achieve functional specificity of the final printed product. Specifically, this technology features the construction of finely arranged or layered microstructures which mimic the microenvironment of cell differentiation and promote targeted maturation of seed cells [168, 169]. Miao's experiments demonstrate that the smart material prepared by this technology has the ability to regulate the proliferation and differentiation of human bone marrow MSCs [170]. This technology may be applied to muscle and nerve tissue engineering in the future.

To summarize, the breakthrough from 3 to 4D bioprinting is a revolution in design technology based on printing materials. By adapting the design strategy for bioprinting materials, 3D biological constructs that change in structure and function over time are ultimately produced. The disciplinary characteristics of plastic surgery are well suited to 4D bioprinting with smart materials. In plastic surgery, wound dressings, auricular implants, nasal septal implants, etc. must be placed in the treatment and surgical area for a prolonged period and sometimes permanently. 4D smart materials have the potential to persist in living organisms for a long time and change structural functions in response to stimulation to adapt to changes in the surrounding environment of the material. The application of 4D bioprinting technology in plastic surgery may be a hot topic for further research. Smart material design may be an effective method to solve the current problems in clinicalization of bioprinting technology.

### Bioprinting for plastic surgery applications

Plastic surgery is the use of surgical methods, or tissue and organ transplantation, to repair and reconstruct defects and deformities of human tissues and organs, as well as to reshape the normal human form to achieve improvement and beautification of form and restoration of function. Plastic surgery involves the repair and reconstruction of a wide range of organ tissues. The problems of infection, pain, and deformity associated with the use of (autologous and allogeneic) grafts in clinical surgery and destructive surgery to the donor area are constantly raising the bar for plastic surgeons. 3D bioprinting not only allows the printing of a variety of different functional cells, ECM, cell growth factors, and biodegradable polymer support materials, but also for personalized and customized implants based on patient needs, including in situ printing directly on the patient's affected area to promote wound repair. The advent of these technologies has greatly reduced the difficulty of surgery, avoided surgical sequelae, and made it possible to achieve true precision medicine [6, 7]. We will highlight the latest research results of 3D bioprinting technology in key areas of plastic surgery, including: skin regeneration and healing, ear reconstruction, rhinoplasty, breast implantation, and maxillofacial bone repair.

#### Skin wounds

Patients with severe burns, diabetic ulcers, tumors, or other etiologies develop severe skin tissue defects and lose the possibility of complete self-regeneration of the skin. Usually, plastic surgeons choose autologous epidermal grafting and autologous flap (bone flap) grafting in clinical treatment according to the patient's skin defect. However, there are still some difficult problems with traditional treatment methods [4, 171, 172]. 3D bioprinting of skin tissue products offers new methods for plastic surgeons to treat skin disorders and promote skin regeneration. GelMA is widely used in skin tissue engineering because of its excellent biocompatibility and light-curing properties. Y Shi et al. proposed the use of a new bioink made of GelMA and COL mixed with tyrosinase to help form the epidermis and dermis [173]. In addition, Lin et al. showed that Si-GelMa, which is made by incorporating Si into GelMA, has a slower degradation rate and enhances the activity of human dermal fibroblasts without reducing its own printability point [174]. Furthermore, Jang et al. also demonstrated the therapeutic effect of GelMA hydrogel containing vascular endothelial growth factor mimetic peptide on wound healing through animal experiments [175]. CHO is also considered to be a biomaterial with great potential in the field of skin repair. Intini's team evaluated CHO's behavior in terms of biocompatibility, cytocompatibility, and toxicity to human fibroblasts and keratin-forming cells through in vitro experiments, demonstrating its great value in the field of skin regeneration [176]. Similarly, Sandri et al. successfully experimentally prepared CHO/glycosaminoglycan-based bioscaffolds for the repair of severe skin lesions, during which they again demonstrated the excellent physicochemical characteristics of CHO materials for skin tissue engineering [177]. Chun-Hsu Yao et al. cross-linked CHO scaffolds with non-toxic genipin and further heparinized it to immobilize the chemokine stromal cell-derived factor-1 (SDF-1) in it. By studying the physicochemical properties and wound healing activity of SDF-1-loaded CHO stents, it was confirmed that SDF-1 therapeutic stents enhanced neovascularization in local wounds and could promote the healing of local skin tissue [178].

**Fig. 5:**
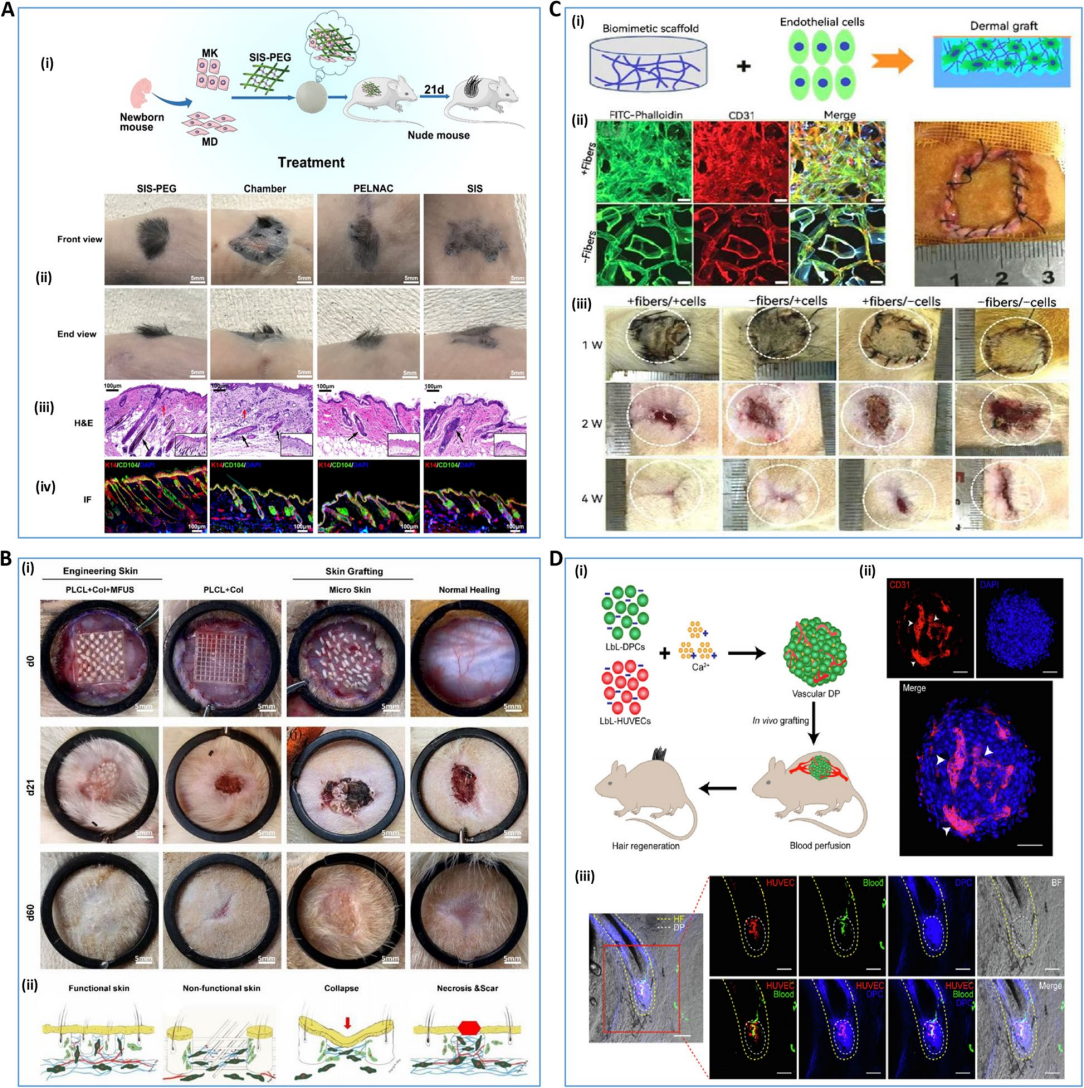
3D bioprinting for trauma repair and hair follicle regeneration **A** SIS-PEG promotes skin wound healing and successful hair growth in mice [179]. (i) schematic diagram of the mechanism, (ii) macroscopic schematic diagram of skin hairs in mice at 21 days, (iii) H&E staining of regenerated skin, (iv) immunofluorescence staining of regenerated skin. **B** PLCL + COL + MFUS (a tissue engineering functional skin by carrying MFUS in 3D-printed polylactide-co-caprolactone scaffold and COL gel) promotes healing of full-thickness skin defect wounds [180]. (i) healing of full-thickness skin wounds in four groups of mice with PLCL + COL + MFUS, PLCL + COL, micro-skin and conventional treatment on days 0, 21 and 60, (ii) schematic diagram of the mechanism of wound healing in each group. **C** macroporous filamentous protein scaffold with nanofibrous microstructures promote neovascularization and dermal reconstruction [182].(i) Schematic diagram of in vivo experiments of endothelial cells-seeded nanofibrous scaffolds, (ii) growth and distribution of seeded cells on the scaffolds, (iii) macroscopic observation of wounds in SD rats at the first, second and fourth weeks after scaffold implantation. **D** layer-by-layer DP spheroids are able to form good blood perfusion in vivo [183] (i) schematic diagram of the mechanism by which layer-by-layer DP spheroids are vascularized in vitro and blood perfusion is formed in vivo, (ii) immunofluorescence showing angiogenesis after 3 days of in vitro culture of layer-by-layer DP, and (iii) immunofluorescence showing blood perfusion after three weeks of in vivo transplantation of layer-by-layer DP. Reprinted with permission from Ref. [179, 180, 182, 183]

Full-thickness skin regeneration remains a difficult clinical challenge. Dong et al. re-cross-linked porcine small intestinal submucosa (SIS) with a four-armed polyethylene glycol (fa-PEG) containing succinimidyl glutarate terminal branches to create a 3D bioactive sponge (SIS-PEG). It has the potential to be an excellent solution to this problem. This study revealed that isolated epidermal and dermal cells loaded with SIS-PEG formed reconstructed skin with regenerated hair after 21 days of treatment [179] (Fig. 5A). Also, Peng Chang introduced the concept of a minimal functional skin unit (MFU): autologous skin with full thickness skin microstructure and complete functional skin unit. They used both nonwoven CHO/ GEL and polylactide-caprolactone COL gel scaffolds to load the MFU. MFU-loaded bioscaffolds exhibit more robust healing ability than those loaded with single seed cells [180] (Fig. 5B). However, lack of vascular architecture, insufficient induction of angiogenesis, and ineffective graft-host anastomosis are major bottlenecks for permanent skin substitutes in tissue engineering. Ma's team successfully synthesized homogeneous strontium silicate micropillars and integrated them into biomaterial inks as stable cell-inducing factors for angiogenesis. They then bioprinting functional skin substitutes based on angiogenesis-induced bionic multicellular systems [181]. In addition, Li seeded endothelial cells derived from human Wharton's Jelly MSCs into a biological scaffold and implanted it into a dermal defect wound of SD rats. This experiment revealed that the multiscale layered design of a macroporous filamentous protein scaffold with nanofibrous microstructures improved the ability of transplanted cells to promote and accelerate neovascularization and dermal reconstitution through enhanced cellular infiltration, COL deposition, and growth factor expression [182] (Fig. 5C).

For patients with severe burns, autologous epithelial grafting is now clinically available for the treatment of burns covering more than 60% of the body. However, although epidermal tissue can be effectively repaired by autologous epithelial grafting, the therapeutic effect on dermal structures that have been ruptured in deep burns is limited. Roshangar et al. isolated adipose-derived stem cells (ADSC) and inoculated them into a 3D gel scaffold made by a 3D bioprinter. They assessed the morphology and cell adhesion properties of 3D scaffolds by hematoxylin–eosin staining and scanning electron microscopy, and determined cell viability by methylthiazolyl diphenyl tetrazolium bromide analysis. Moreover, an experimental treatment observation on a rat model of whole-layer burns revealed that 3D gel scaffolds with or without ADSC accelerated wound contraction and healing. However, rats treated with gel scaffolds prepared with bioink loaded with ADSC exhibited earlier epithelialization [184]. 3D bioprinting technology combined with stem cells may be the focus of future research aimed at achieving the healing of severe skin lesions.

In addition to basic skin tissue repair, the repair of skin-related appendages is also an important research direction in the field of regenerative medicine, especially for the treatment of hair loss, which has received widespread attention. The biggest challenge for hair follicle reconstruction and repair is to maintain the hair growth-inducing properties of dermal papilla cells (DPCs). Chen et al. used layer-by-layer self-assembly of GEL and Alg to construct Nano-biomimetic ECM of DPCs. They used Ca^2+^ as a cross-linking agent and created controllable vascularized dermal papilla (DP) spheroids by co-culturing DPCs with human umbilical vein endothelial cells. The results showed that the controlled DP spheroids made by this method were highly similar to native DP spheroids (Fig. 5D). It was also found that nanoscale ECM and vascularization restored the transcriptional characteristics of post-transplant DPCs and tripled the efficiency of hair induction compared to conventional 3D culture [183]. In addition, Zhang et al. constructed a skin model with sweat glands and hair follicles through bioprinting technology. In an observational study of this model, it was found that hair follicle spheroids promoted the differentiation of sweat glands and hair follicles, while sweat gland scaffolds promoted sweat gland differentiation, but had little effect on hair follicle potency in hair follicle spheroids [185]. Current studies on hair follicle regeneration are all in vitro at the cellular level, and there is a lack of reliable animal studies to demonstrate the possibility of hair follicle regeneration. In the future, animal experiments on hair follicle regeneration to demonstrate the feasibility of in vitro culture of hair follicles may be a key step to solve the problem of hair follicle regeneration research from the laboratory to the clinic. Therefore, this research deserves the attention and exploration of the scholars.

#### Ear reconstruction

**Fig. 6:**
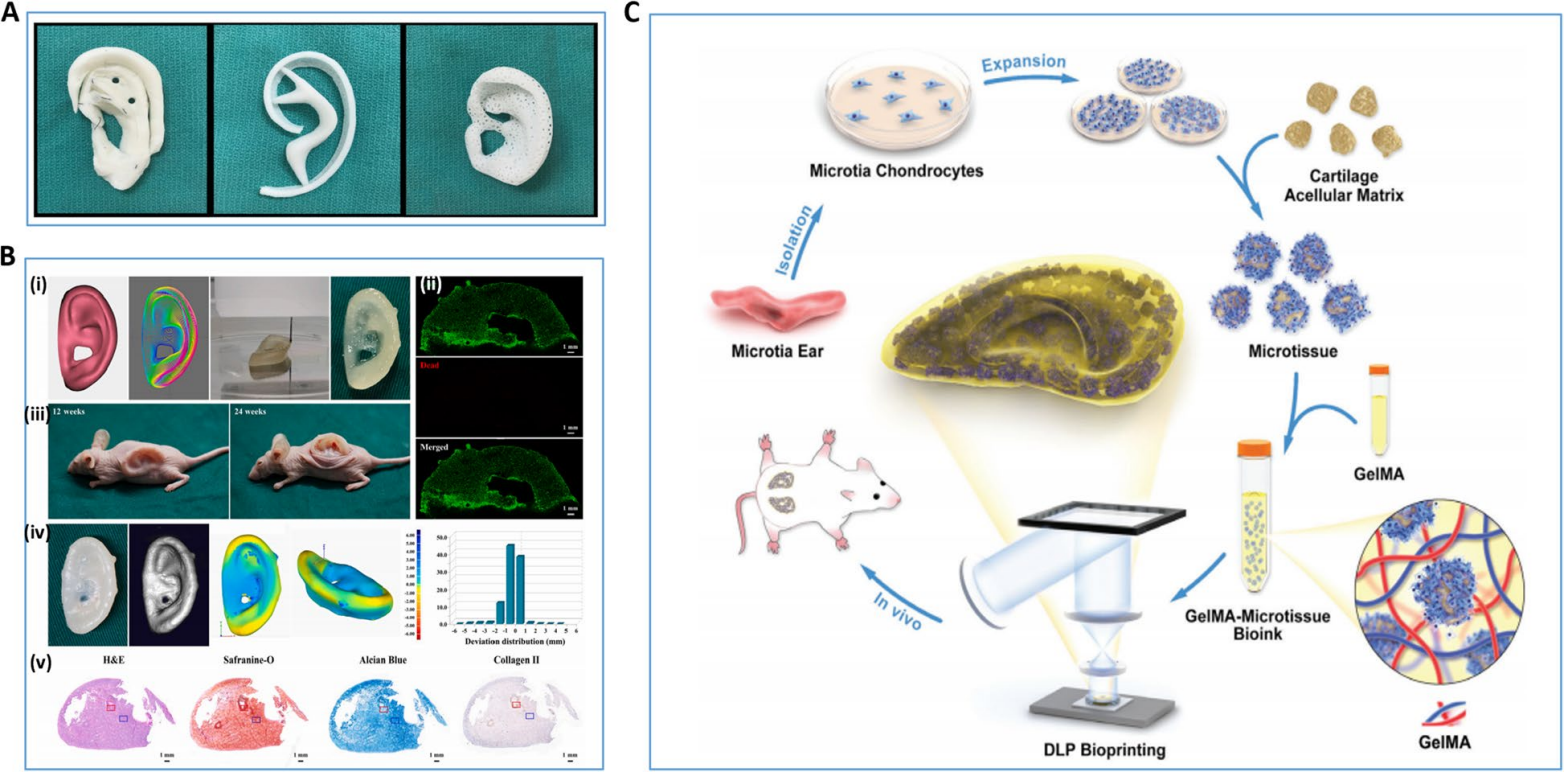
3D bioprinting for ear reconstruction. **A** Demonstration of three methods of preparing ear cartilage for ear reconstruction, i.e., traditional surgical method, 3D bioprinting ear cartilage, and 3D bio-scaffold [8]. **B** 3D bioprinting produces pinna cartilage [187]. (i) 3D digital model of the human ear and bioprinting ear structures based on biomimetic microporous methacrylate-modified acellular cartilage matrix microporous bioink, (ii) live/dead cell staining of bioprinting ear structures, (iii) well-preserved in vivo regenerated ear cartilage structures in nude mice after 12 and 24 weeks of culture, (iv) 3D modeling bias present in the regenerated ear cartilage, (v) H&E, saffronin-O, Alcian blue and COL II staining of the regenerated ear cartilage after 24 weeks of in vivo culture. **C** Schematic representation of preparation of microtissue bioink and its application to Digital Light Processing bioprinting [188]. Reprinted with permission from Ref. [8, 187, 188]

The ear has important socio-cultural, aesthetic and functional value, and patients with congenital and acquired ear deformities often bear a great deal of psychological stress. The use of fine autogenous rib cartilage has been the gold standard for ear reconstruction for the past half century. However, the ear reconstruction procedure requires a high level of surgical and artistic skill in obtaining and sculpting the patient's rib cartilage. This ensures that a beautifully reconstructed auricular framework is obtained after the rib cartilage is inserted into the skin pocket in the area of the deformed ear. The introduction of 3D bioprinting technology can greatly reduce the difficulty of surgery and make it possible to customize molds for patients [8, 9] (Fig. 6A). Simply put, this technology first uses DICOM CT images to recreate the external anatomy of the human ear and adjust it to the existing design, then selects the appropriate biomaterial ink or bioink to 3D print the appropriate ear cartilage product [8]. In 2018, the team of Zhou et al. achieved the first international clinical breakthrough with a tissue-engineered ear made from polyglycolic acid/polylactic acid and chondrocytes [186]. However, the postoperative deformation and inflammation demonstrated the immaturity of the technique. In 2022, Jia et al. from the same team proposed a new approach to address the inflammatory response and structural deformation that can occur in reconstructed structures. They used bioactive bioinks based on auricular chondrocytes and biomimetic microporous methacrylate modified decellularized cartilage matrix with the aid of GelMA, polyethylene oxide and PCL to prepare biologic auricular structures with precise shape, low immunogenicity and excellent mechanical properties using integrated multi-nozzle bioprinting technology [187] (Fig. 6B).

Brennan's team has explored a different method of 3D ear cartilage printing. They printed multiple auricular scaffold products using laser sintering PCL and implanted each under the skin of thymus-free rats. The researchers monitored the rats weekly for ulcer formation, infection, and stent deformation in the surgical area. The stents were removed at week 8 and analyzed using micro computed tomography and histological staining. The auricular scaffold they designed and fabricated demonstrated excellent implantation ease, appearance, vascularization, and acceptable superficial wound complication rates in animal models [189]. In addition, co-culture technology is expected to be an effective way to solve the scarcity of auricular chondrocytes in 3D bioprinting. Dong et al. co-cultured auricular chondrocytes with human MSCs at 10/90, 25/75, and 50/50 ratios for 6 months. After several observations, it was possible to obtain structurally well maintained and healthy human elastic cartilage by this method [190]. Posniak et al. chose to use a 3D bioprinting scaffold made from a combination of GelMA and methacrylic acid-hyaluronic acid. They used this scaffold to assist in detecting differences in the results of monoculture and co-culture of human septal chondrocytes (primary chondrocytes, [PC]) and human bone marrow MSCs. The results showed that the co-culture combination of MSCs and PCs exhibited not only cell proliferation mimicking MSCs, but also chondrogenic expression mimicking PCs [191]. These studies represent the possibility of preparing large quantities of elastic cartilage in vitro, marking a key step in the translation of auricular tissue engineering to the clinic.

Enhancing the survival rate of auricular chondrocytes is still a challenge to be solved in cartilage tissue engineering. Xie's team designed a method to cross-link decellularized matrix particles with GelMA hydrogels to produce a micro-tissue bioink. This microtissue bioink has ideal mechanical properties and swelling rates with little effect on printability. The team also produced fine auricular structures using microtissue bioink based on residual ear cartilage cells. The residual ear chondrocytes in the printing products showed excellent performance in both in vitro cell proliferation and in vivo ear cartilage regeneration. The main reason is that this micro-tissue composite bioink, not only can accurately assemble organ building blocks, but also provides a 3D refuge for the cells to ensure the viability of the printed cells [188] (Fig. 6C). This technology has greatly enhanced the proliferation and differentiation of chondrocytes in 3D auricular constructs and is expected to be widely investigated and applied in other areas of cartilage tissue engineering.

#### Rhinoplasty

**Fig. 7:**
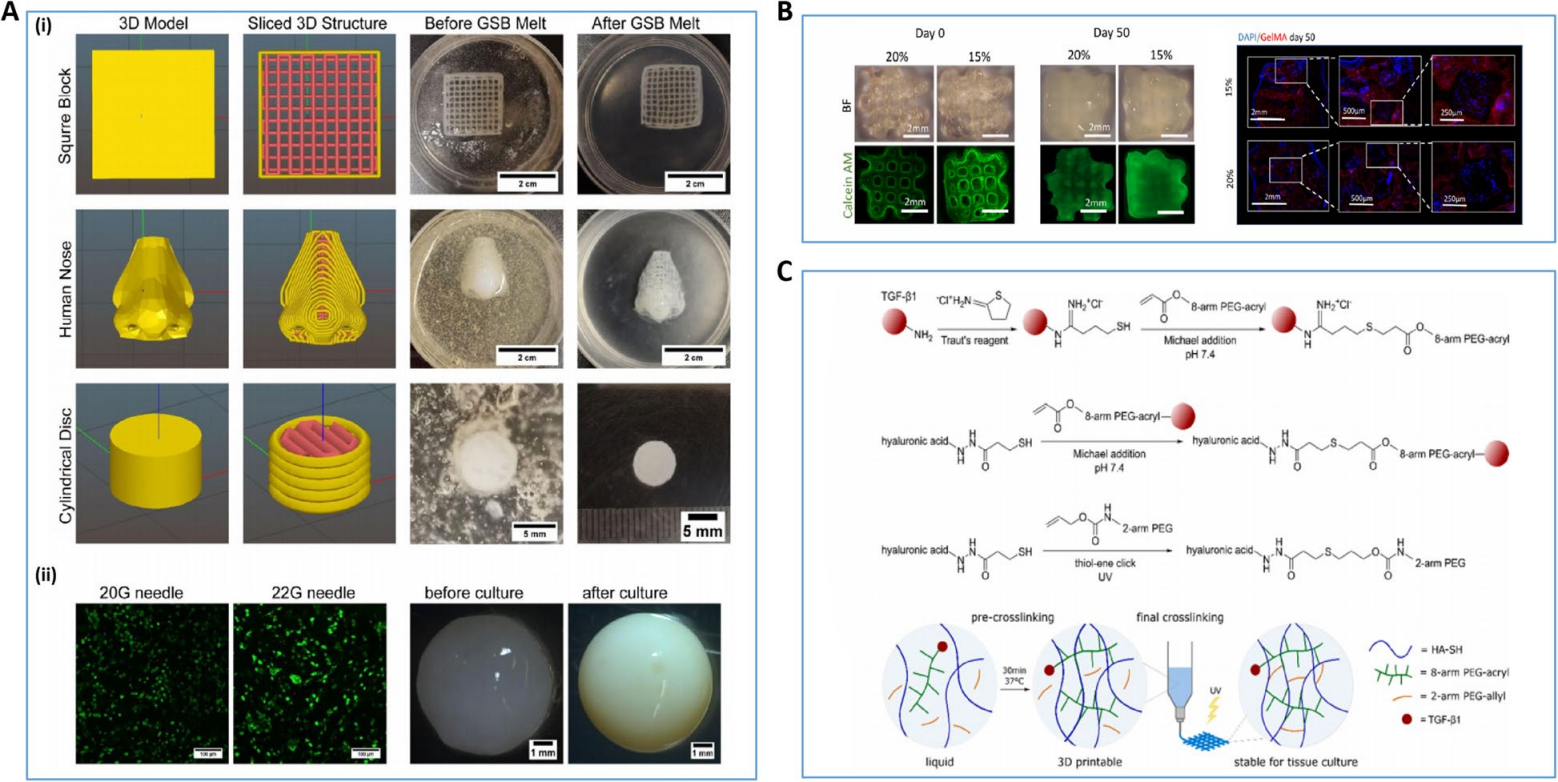
3D bioprinting for Rhinoplasty. **A** (i) 3D digital models of square blocks, human noses and cylindrical shapes after importing Slic3r and 3D bioprinting products, (ii) Detection of chondrocyte activity after 3 days of culture by live/dead with 20G and 22G needles, (iii) Alterations in the macroscopic structure of freeform reversible embedding of suspended hydrogel bioprinting constructs before culture and after 6 weeks of culture [192]. **B** (i) Macrostructure of GelMA/chondrocyte print products based on day 0 and day 50 with calcein AM staining of cell fluorescence images (ii) Day 50, cell distribution of frozen sections using DPI staining showing GelMA/chondrocyte structure [196]. **C** Schematic diagram of the cross-linking mechanism of the bioink with transforming growth factor-β1 [197]. Reprinted with permission from Ref. [192, 196, 197]

Due to tumor, injury, and the patient's own aesthetic needs, plastic surgeons need to reconstruct patient nasal cartilage. However, because nasal cartilage lacks the ability to repair itself well, current rhinoplasty usually uses autologous cartilage or synthetic implants for therapeutic and cosmetic surgical purposes. Autologous cartilage is considered the best choice of graft due to its low immune rejection. As with the ear reconstruction mentioned above, rhinoplasty is considered one of the most challenging plastic surgery procedures due to the high manual and artistic skill required of the surgeon during the procedure. Lan et al. investigated the effect of culture time on the ECM formation and mechanical properties of 3D bioprinting structures of type I COL hydrogels loaded with human nasal chondrocytes in vitro and in vivo. Experiments demonstrate that 3D bioprinting nasal cartilage structures are a viable option for rhinoplasty [192] (Fig. 7A). 3D bioprinting technology promises to significantly reduce the surgical difficulty of rhinoplasty making it possible to customize individual nasal cartilage structures. Specifically, the process of this technique is similar to the 3D bioprinting technique used in ear reconstruction. It also prints customized nasal cartilage models by precisely depositing bioink using patient image data and the assistance of a computer. Interestingly, the teams of Choi et al., Suszynski et al., and De Greve et al. were able to simulate the post-surgical nasal appearance of the patient and the post-surgical nasal cartilage model prior to surgery through 3D modeling and 3D printing techniques. Plastic surgeons can use these mimetic models as a reference to be able to greatly reduce the difficulty for the surgeon and improve patient satisfaction [193–195]. Although these techniques are valuable in terms of 3D bioprinting technology and actual surgical operations, the 3D structural models printed do not use active bioprinting materials. Therefore, they are not considered 3D bioprinting techniques.

Ruiz-Cantu's team experimentally investigated the effects of temperature, needle distance, UV exposure time, and cell carrier formulation (GelMA) on the survival and functionality of chondrocytes in bioprinting constructs. GelMA at 20% w/v was found to be the optimal concentration for 3D bioprinting of chondrocytes. After a 50-day culture period, the 3D bioprinting constructs showed neochondral formation and mechanical properties similar to those of nasal cartilage. This study confirms the feasibility of using chondrocyte/GelMA/PCL bioinks for printing nasal cartilage structures [196] (Fig. 7B). In addition, the effect of the microenvironment of the bioink on the chondrocytes also greatly affects the quality of the final printed product. The team of Su-Shin Lee et al. used a supercritical carbon dioxide technique to extract decellularized porcine nasal cartilage (dPNCG). They also developed and constructed a bioactive 3D tissue-based construct consisting of different ratios of ADSC, chondrocytes, and dPNCG. Their study confirmed that dPNCG is an excellent matrix scaffold that provides a suitable microenvironment for chondrocytes and is capable of printing suitable nasal cartilage structures [198].

In cartilage tissue engineering, covalent binding of growth factors to printable bioinks is a challenge that remains unconquered. Hauptstein et al. constructed a two-stage cross-linked hyaluronic acid-based bioink capable of binding growth factor β1 through covalent bonding. The bioink composition produces higher quality cartilage tissue which does not require a continuous supply of exogenous growth factors [197] (Fig. 7C). Based on the advantages of this bioink composition to form printed biological tissue capable of growth and development, it may also be applied to future areas of tissue engineering research.

#### Breast implants

As people's aesthetic requirements continue to change, the number of patients with breast augmentation requirements is increasing year by year. There are two main types of breast augmentation: breast prosthesis implantation and autologous fat implantation. However, both of these surgical procedures have varying degrees of drawbacks. Autologous fat implantation, simply put, is the transplantation of the patient's own fat tissue into the breast organ. However, due to the early inflammatory factors formed in the grafted fat tissue in the breast and the ischemic and hypoxic environment of the fat tissue, adverse surgical sequelae such as fat resorption, fat necrosis, cavity formation, and tissue fibrosis occur. In contrast, breast prosthesis implantation can achieve breast augmentation in a safer and more convenient way. However, the problems of implant envelope formation, stiffness and late infection make it difficult to fully satisfy the aesthetic needs of patients [2, 199]. The teams of Dilyana Todorova et al. and Shan Mou et al. proposed the use of EVs of ADSC to assist in the transplantation of adipose. However, this technique is not yet mature due to factors such as hypoxic time of adipocytes, insufficient angiogenesis and limited ability of nutrients to penetrate tissue fluid [200]. The advent of 3D bioprinting technology raises the possibility of growing a mature vascularized breast-like adipose tissue structure outside the body through 3D bioprinting technology and then implanting the structure into the patient's breast.

**Fig. 8:**
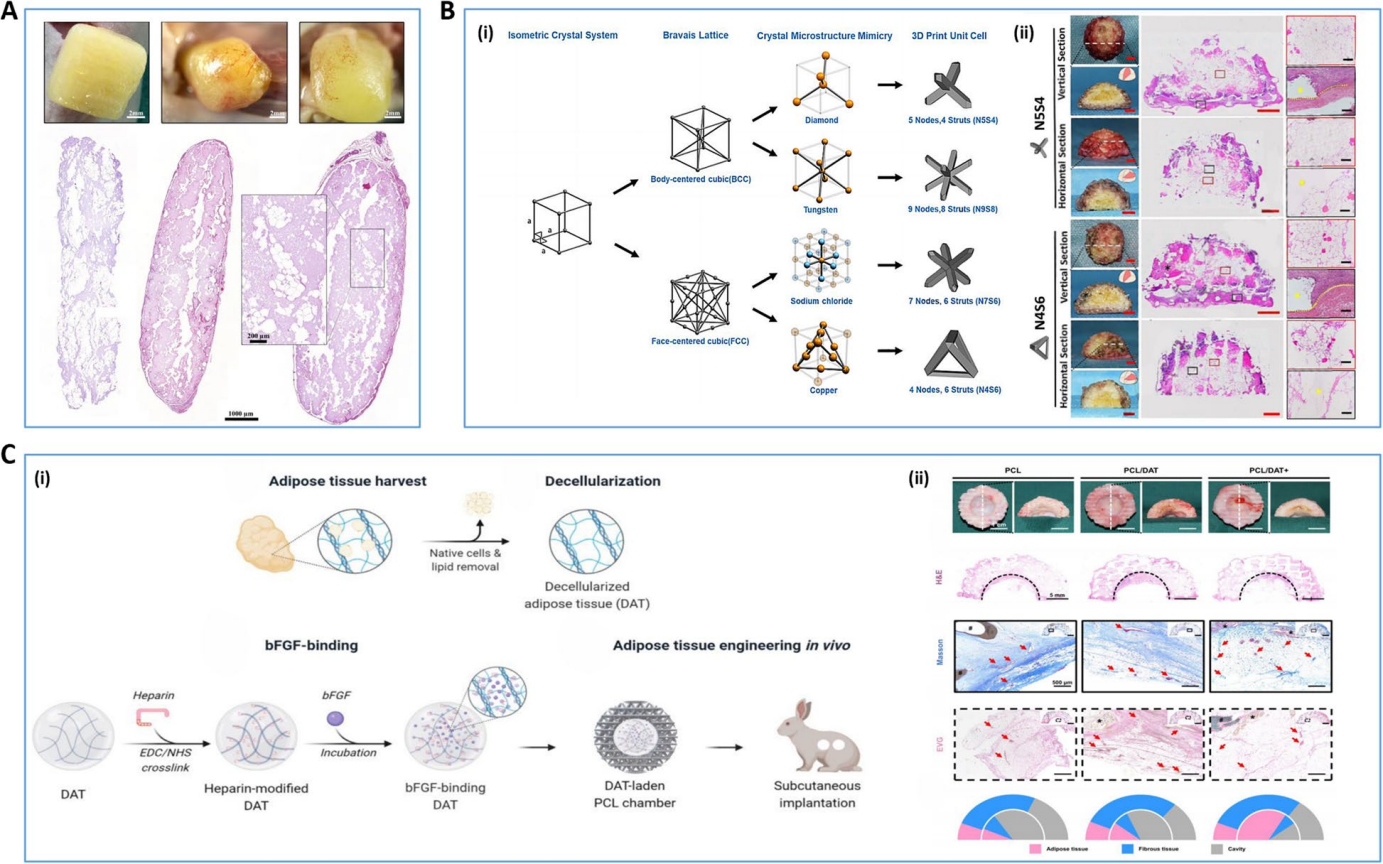
3D bioprinting for Breast Implants **A** Histological images and macroscopic images of freshly printed 3D bioprinting LAT products and 3D bioprinting LAT products cultured in vivo for 30 days and 150 days [202]. **B** The survival rate of fat in different breast scaffolds was different in nude mouse model experiments [203]. (i) Schematic design of crystal microstructure of unit cells. (ii) After 12 weeks of fat grafting, comparing macroscopic images and H&E staining images of N5S4 and N4S6 groups, the adipose tissue in the N5S4 group had a more regular shape and better integration of the scaffold with adjacent tissues, with less compression. **C** The Macroporous chambers facilitate large volume soft tissue regeneration from adipose-derived extracellular matrix [204]. (i) Mechanisms to promote the generation of large soft tissue volumes in the ECM of adipocytes using large pore chambers, (ii) Morphological performance of grafted specimens from the PCL, PCL/DAT, and PCL/DAT + groups after 12 weeks, with PCL/DAT + showing better vascularization and more adipose tissue generation. Reprinted with permission from Ref. [202–204]

Tong's team first demonstrated the ability of ADSCs to transform into epithelial-like cells through in vitro 3D culture experiments, confirming the positive effect of ADSC on adipose metastasis [201]. In addition, Saljo et al. explored the long-term in vivo viability of 3D bioprinting lipoaspirate-derived adipose tissue (LAT) and its proteomic profile and cellular composition. Experiments have demonstrated that LAT has a good proteomic profile and that its cellular components, including 3D bioprinting adipose ADSC, endothelial progenitor cells and blood vessels, can survive for a long time (Fig. 8A). This result reaffirms the feasibility of 3D bioprinting in adipose tissue engineering [202]. However, the survival rate of adipose tissue in 3D bioscaffolds is still a challenge. Zhou et al. designed and manufactured four types of breast scaffolds using polyurethane. The basic unit cell of each scaffold resembles the lattice structure of an isometric crystal system, and each scaffold has the same porosity, but different mechanical properties. Experiments with a nude mouse model revealed that adipose survival was higher in scaffolds (N5S4) possessing a similar compression modulus to natural breast tissue, and vascularization and mild fibrosis could be observed (Fig. 8B). This lattice-like structural design has led to a further expansion of the study of adipose tissue engineering in the breast [203]. In addition, large volume adipose tissue generation is still a technical challenge. Tissue engineering chambers (TEC) are considered to be an effective technique for generating large volumes of adipose tissue. However, the application of TEC requires reoperation to remove the non-degradable plastic cavity and excise some of the autologous tissue, which greatly hinders its practical use in the clinical setting. To deal with this problem, Zhang et al. devised an improved TEC strategy combining a bioresorbable PCL chamber structure and decellularized adipose tissue (DAT). They prepared a microporous PCL chamber structure and prepared DAT containing basic fibroblast growth factor (bFGF). In a rabbit experimental model, highly vascularized adipose tissue that nearly filled the PCL lumen (5 mL) was regenerated from DAT loaded with 0.5 mL of bFGF. The newly formed tissues had significantly higher expression of adipose genes compared to endogenous adipose tissue in the control group [204] (Fig. 8C). The results of this experiment make it possible to generate large volumes of adipose tissue in vitro using 3D bioprinting technology, and are expected to be applied to clinical treatments and other adipose tissue engineering studies in the future.

#### Maxillofacial bone restoration

The maxillofacial region is a complex area composed of multiple tissues, including maxillofacial bone, skeletal muscles, gums, and periodontal ligaments. When tissue is lost in the maxillofacial region due to tumors, trauma, and other pathological factors, the patient is often restored using autologous, allograft, or xenograft surgery, and often requires joint treatment by oral, maxillofacial, and plastic surgery [1, 205–207]. Since the applications of 3D bioprinting technology in soft tissue and cartilage tissue engineering, etc. have been described above, we now will only review the application of 3D bioprinting technology in the field of plastic surgery for maxillofacial bone tissue repair.

**Fig. 9:**
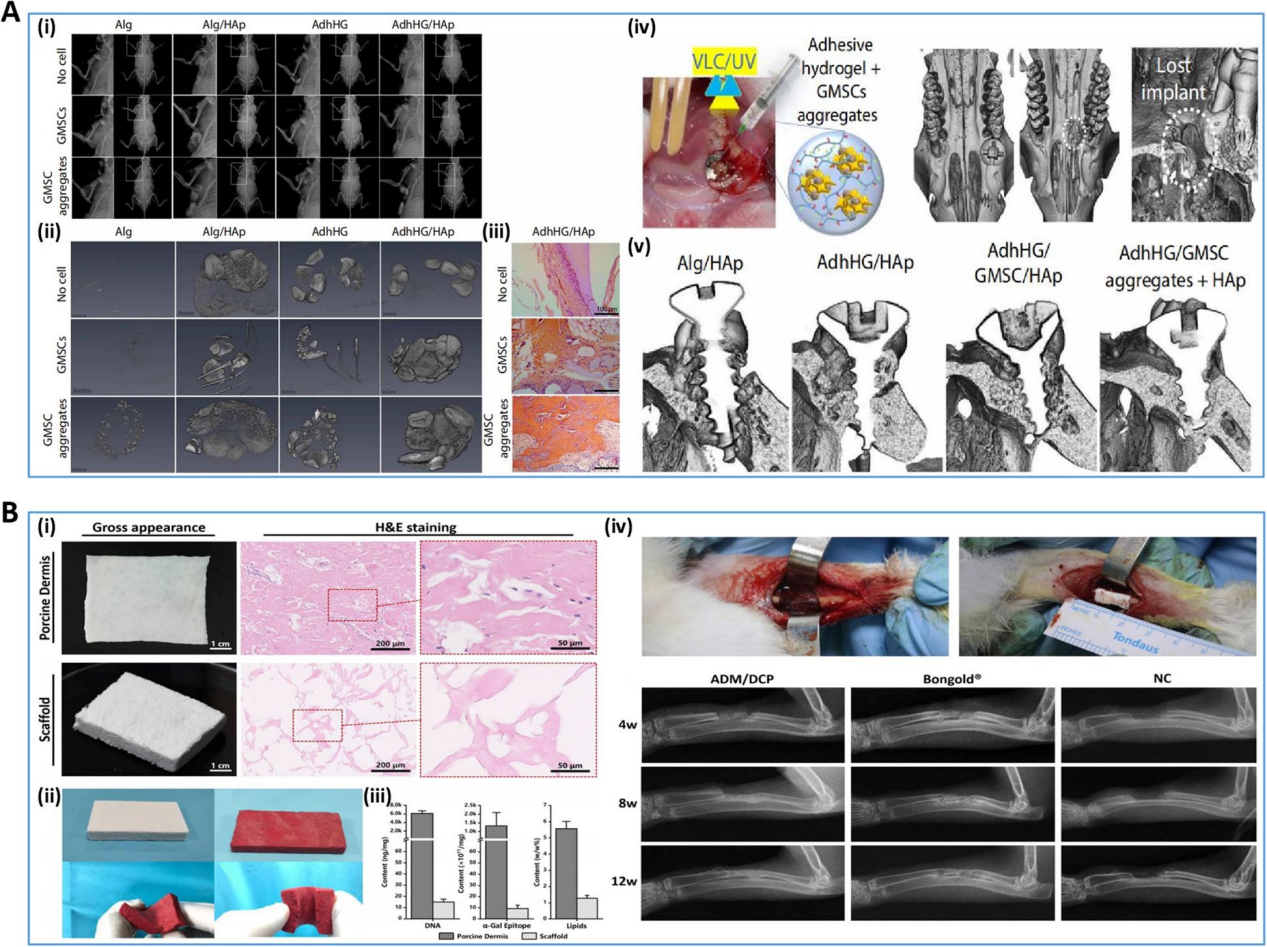
3D bioprinting for Maxillofacial Bone Restoration. **A** Experimental demonstration of bone regeneration capacity of Alg-based adhesive hydrogel (AdhHG)/ Gingival MSCs (GMSC) aggregates + hydroxyapatite (HAp) [213]. (i) Four groups of Alg, Alg/HAp, AdhHG and AdhHG/HAp were mixed with cell-free formulations, GMSC and GMSC aggregates to prepare different hydrogels for implantation under the skin, respectively, and 2D radiographs were observed, (ii) 3D reconstructed images of each group of CT imaging, (iii) H&E staining pictures of each group after 8 weeks of subcutaneous implantation of hydrogels mixed with GMSC, GMSC aggregates, or cell-free formulations of AdhHG/HAp, (iv) Actinomycetes-coated titanium implants cause peripheral inflammation and defects, hydrogels mixed with different formulations are injected into oral defects, (v) 8 weeks after implantation of hydrogels, showing that hydrogels containing different formulations promote regeneration [213]. **B** Performance demonstration of 3D porous scaffolds based on dicalcium phosphate decellularized dermal matrix [215]. (i) macroscopic appearance and H&E staining of porcine dermis and prepared scaffold to reflect their nucleus-free components, (ii) blood absorption performance of scaffold, (iii) DNA, a-Gal epitope and lipid content display of porcine dermis and scaffold, (iv) X-ray schematic diagram of the middle bone defect of the middle radius in the Dermis-derived matrix/dicalcium phosphate, and negative control group at the 4th, 8th and 12th weeks after surgery, to compare the ability to promote bone regeneration. Reprinted with permission from Ref. [213, 215]

Specifically, the clinical treatment of bone defects in the maxillofacial region is usually dictated by the condition of the maxillofacial bone defect. Since the periosteum has the ability to regenerate and differentiate into osteogenic bone, it is possible to repair small maxillofacial defects using only the periosteum method [208, 209]. For large maxillofacial defects, a combination of seed cells (mostly bone marrow-derived MSCs or ADSC) and a biological scaffold is commonly used for treatment [210]. Bioactive components are also often added to biological scaffolds in some studies to promote proliferation and differentiation of stem cells [205, 211]. Unlike conventional bone tissue engineering studies, Shie et al. prepared a 3D porous bioceramic scaffold by combining two printing techniques. They first prepared ceramic scaffolds using extrusion-based bioprinting, and then printed stem cells directly onto the surface of the ceramic scaffolds using a piezoelectric nozzle. They also verified the hydrophilicity and cell adhesion of polydopamine calcium silicate/polycaprolactone using controlled experiments. Since the printing technology of piezoelectric nozzles can print cells more precisely, it is expected to be used to repair complex bone tissue in the maxillofacial region [212]. However, the use of rigid, solid 3D scaffolds inevitably makes it difficult to adapt to the complex bone tissue structure of the maxillofacial region. Therefore, scaffolds that can inject semi-solid and or gel-like materials directly into the defect have become a more practical and promising design. The research team of Hasani-Sadrabadi et al. designed an Alg-based osteoconductive hydrogel biomaterial with high adhesive capacity, photocrosslinkable and tunable mechanical properties. They demonstrated its good biodegradability, biocompatibility, and osteoconductivity as well as complete maxillofacial bone repair in mouse experiments [213] (Fig. 9A). In addition, COL gels have been shown to better promote soft tissue healing around bone defects, and a study by Salamanca et al. showed that collagenized porcine grafts promote better bone regeneration and reduce bone loss [214]. In recent years, the variety of bone graft substitutes for maxillofacial bone remains limited, which hinders the development of maxillofacial bone tissue engineering. Li et al. successfully prepared porous scaffold structures using skin-derived matrices (ADM) with the aid of micronization techniques. Then, they prepared composite scaffolds with high porosity and interconnected pores by incorporating dicalcium phosphate particles into ultrafine ADM fibers and freeze-drying them to form highly porous structures [215] (Fig. 9B). This new bone graft substitute is expected to be further investigated in maxillofacial bone tissue repair.

## Challenges of 3D bioprinting

After decades of development, 3D bioprinting has come a long way. However, there are still many challenges in the application of 3D bioprinting technology in burn patients. On a technical level: 1) Plastic surgery patients have a higher aesthetic demand for improved and repaired tissues and organs, so the 3D model reconstructed from the collected data must be accurate, and even slight deviations may lead to surgical failure. Specifically, in plastic surgery, the main focus is on bone, cartilage and soft tissue repair. The main methods available for data acquisition are still dominated by CT and MRI imaging techniques, and methods that enable more accurate data acquisition deserve further exploration [216–218]. 2) Although some simple 3D printing strategies and bioprinting material designs have been proposed with the continuous changes in printing technology, the cost of 3D bioprinting is still very high and requires a high level of operator skill during the entire process. 3)Plastic surgery is a discipline involving the repair of multiple tissues and organs, and the printing strategies and bioprinting materials used for different tissues and organs are bound to be different, which undoubtedly increases the technical requirements of the staff in this discipline. 4) At present, there is still a gap between 3D bioprinting products and clinical practical application, especially full-thickness skin repair, skin follicle repair, with vascular function printing products and other aspects of research awaiting technical breakthroughs. On an ethical level, the source, quality, and safety of cells and materials in 3D bioprinting, as well as the ethical discussion of animal and human experimentation on printed products, are also essential professional questions for those working in the bioprinting field [219].

## Conclusions

In conclusion, 3D bioprinting technology allows personalized 3D printed products to be finely tailored to the patient's needs, and avoids the surgical complications and adverse reactions that can occur in traditional surgery. In addition, 4D bioprinting technology can enable printing products to change over time. This biological printing product that changes its shape or function over time provides a new idea for the tissue repair and beautification of plastic surgery. Moreover, 3D bioprinting can produce bioprinting products that meet different needs by selecting different bioprinting technologies and bioprinting material strategies. These printing products have been applied to the repair of skin, ear cartilage, nasal cartilage, maxillofacial bone, etc. in the field of plastic surgery, and show good therapeutic potential. From printing the simplest scaffold bionic structures to 3D bioprinting in practice clinical application, one breakthrough and change in 3D bioprinting technology symbolizes the infinite possibilities of life sciences. However, 3D bioprinting technology still has a long way to go from the laboratory to the clinic, and there are still some difficulties and challenges to face and solve on this road. Only through the continuous efforts of many researchers can 3D bioprinting technology widely used in the clinic to meet the needs of patients.

## Data Availability

Not applicable.

## Abbreviations

3D: Three-dimensional
DICOM: Digital imaging and communications in medicine
CT: Computed tomography
MRI: Magnetic resonance imaging
STL: Standard tessellation language
SLA: Stereo lithography appearance
LAB: Laser-assisted bioprinting
UV: Ultraviolet
DOD: Drip-on-demand
ECM: Extracellular matrix
ployP: Polyphosphate
MSCs: mesenchymal stem cells
PCL: polycaprolactone
PLA: polylactide
PU: polyurethane
dECM: decellularized ECM
COL: collagen
GEL: gelatin
Alg: alginate
RGD: arginine-glycine-aspartic acid
GelMA: methacrylate gelatin
HA: hyaluronic acid
SF: silk fibroin
CHO: chitosan
EVs: extracellular vesicles
MSCs: mesenchymal stem cells
GF: growth factor
MFU: minimal functional skin unit
PAA: poly (acrylic acid)
PVDT: poly (2-vinyl-4,6-diamino-1,3,5-triazine)
PBS: phosphate buffered saline
PEG: poly (ethylene glycol)
SIS: small intestinal submucosa
ADSC: adipose-derived stem cells
DPCs: dermal papilla cells
DP: dermal papilla
PC: primary chondrocytes
dPNCG: decellularized porcine nasal cartilage
LAT: lipoaspirate-derived adipose tissue
TEC: tissue engineering chambers
DAT: decellularized adipose tissue
bFGF: basic fibroblast growth factor
AdhHG: Alg-based adhesive hydrogel
GMSC: gingival MSCs
